# Neuron-specific WDR5 epigenetically upregulates ARID5B to impair GABAergic synaptic transmission and promotes epileptogenesis

**DOI:** 10.7150/thno.122246

**Published:** 2026-01-01

**Authors:** Juan Gu, Ping-Yang Ke, Xin-Yu Zhang, Chao Liu, Yuan Yang, Ming-Lan Yu, Zhen-Zhong Xu, Chun-Xiang Zhang, Wei Dong

**Affiliations:** 1 Key Laboratory of Medical Electrophysiology of Ministry of Education and Medical, Electrophysiological Key Laboratory of Sichuan Province, Institute of Cardiovascular Research, Southwest Medical University, Luzhou 646000, China.; 2 Department of Neurology, The Affiliated Hospital of Southwest Medical University, Luzhou 646000, China.; 3 Department of Anesthesiology, First Affiliated Hospital and School of Brain Science and Brain Medicine, Zhejiang University School of Medicine, Hangzhou 310058, China.; 4 Department of Neurology, Chongqing Key Laboratory of Major Neurological and Mental Disorders, Neurology Key Laboratory of Chongqing Education Commission of China, Chongqing Key Laboratory of Neurology, The First Affiliated Hospital of Chongqing Medical University, Chongqing 400016, China.

**Keywords:** ARID5B, epilepsy, GABAergic synaptic transmission, H3K4me3, WDR5

## Abstract

**Rationale:** Temporal lobe epilepsy (TLE) is a prevalent, drug-resistant neurological disorder that causes severe disability, highlighting the need to identify novel therapeutic targets. Emerging evidence reveals widespread transcriptional dysregulation during epileptogenesis, in which multiple dysregulated genes functionally contribute to disease progression. However, the epigenetic basis of these transcriptomic changes remains poorly characterized.

**Methods:** We established experimental epilepsy models and systematically investigated the remodeling of major histone methylation modifications during epileptogenesis using integrated low-throughput molecular assays and epigenomic profiling. Through pharmacological and genetic interventions, combined with synchronized video-EEG monitoring, whole-cell patch-clamp recordings, multi-omics analyses, and *in vivo*/*ex vivo* molecular biology approaches, we mechanistically dissected the pathological role of elevated histone H3 lysine 4 trimethylation (H3K4me3) in epileptogenesis.

**Results:** We demonstrated the dynamic increase in H3K4me3, an epigenetic marker of transcriptional activation, during TLE development. Both pharmacological inhibition of the WD repeat-containing protein 5 (WDR5)-lysine methyltransferase 2 (KMT2) methyltransferase complex and neuron-specific WDR5 knockdown consistently conferred anti-epileptogenic effects. Mechanistically, integrated ChIP-seq and RNA-seq analyses identified AT-rich interactive domain-containing protein 5B (ARID5B) as a key WDR5-targeted effector in hippocampal neurons. WDR5 enhances H3K4me3 deposition at the *Arid5b* promoter to drive its transcriptional upregulation. The upregulated ARID5B protein subsequently represses γ-aminobutyric acid type A receptor (GABA_A_R) subunit expression, impairing inhibitory synaptic transmission and facilitating epileptogenesis. In addition, the WDR5-H3K4me3 axis may directly or indirectly regulate genes involved in glutamatergic synaptic transmission.

**Conclusions:** The neuronal WDR5-H3K4me3 axis is an important epigenetic driver in epileptogenesis, providing both mechanistic insights and promising therapeutic targets for early intervention.

## Introduction

Epilepsy is a chronic and disabling neurological disorder, with a global prevalence of active epilepsy estimated at approximately 51 million individuals 1, 2. The mechanism of temporal lobe epilepsy (TLE), the most prevalent epilepsy syndrome in adults, remains unclear, and TLE is often refractory to the current available antiseizure medications (ASMs) 3, 4. Multitarget drug therapy has emerged as an important therapeutic strategy for managing complex diseases 5. Emerging evidence highlights the pivotal role of epigenetic mechanisms in epileptogenesis 6. Compared with conventional ASMs, epigenetic modulation offers unique advantages for epilepsy treatment through its inherent ability to act on multiple targets to combat complex pathological changes 7, 8. Notably, epigenetic drugs have demonstrated manageable safety profiles, with substantial preclinical evidence supporting their applications in neurological disorders 8, 9. Therefore, restoring epigenetic homeostasis represents a promising therapeutic strategy for epilepsy.

Histone methylation is a highly conserved and well-characterized epigenetic modification that serves as a critical upstream regulator of transcription, with established physiological and pathological significance 10, 11. The transcriptional effects of histone methylation depend on both the specific modified residue and the degree of methylation at each genomic locus 12. Notably, the methylation of histone H3 lysine 4 (H3K4) is generally associated with transcriptional activation 13. While H3K4 trimethylation (H3K4me3) predominantly occurs in promoter and coding regions, H3K4 monomethylation (H3K4me1) typically occurs in enhancer elements 13. In contrast, the trimethylation of H3 lysine 9 or 27 (H3K9me3, H3K27me3) is a repressive histone mark associated with heterochromatin 14, 15. The homeostasis of histone methylation is dynamically regulated through the opposing activities of histone methyltransferases (including lysine methyltransferase 2 [KMT2] family proteins) and histone demethylases (such as lysine-specific demethylases 5A-D [KDM5A-D]) 13, 16. The KMT2 family (SETD1A/B, KMT2A-D) requires assembly with WD repeat-containing protein 5 (WDR5), Set1/Ash2 histone methyltransferase complex subunit ASH2 (ASH2L), retinoblastoma-binding protein 5 (RBBP5) and protein dpy-30 homolog (DPY30) - hereafter referred to as the WDR5-KMT2 complex - for catalytic activity 16. Genetic studies link mutations in these genes to autism spectrum disorders, refractory epilepsy and other neurodevelopmental abnormalities 17-19. Moreover, experimental evidence supports the role of histone methylation in epilepsy. For example, Zhang et al. demonstrated increased potassium inwardly-rectifying channel, subfamily J, member 10 (*Kcnj10*), in pilocarpine-induced epileptic rats, potentially via H3K9me2-dependent transcriptional regulation 20.

Transcriptional dysregulation of numerous genes occurs during epileptogenesis 4, 21-23. However, the potential involvement of histone methylation—a key epigenetic mechanism that governs gene expression—in the modulation of epilepsy-associated gene networks remains systematically unexplored. Here, we report dynamic H3K4me3 remodeling in TLE. We identified a neuron-specific WDR5-H3K4me3-AT-rich interactive domain-containing protein 5B (ARID5B) epigenetic axis that mediates the downregulation of γ-aminobutyric acid type A receptors (GABA_A_Rs), thereby disrupting the excitation-inhibition (E/I) balance to promote epileptogenesis. Therapeutic targeting of this axis has potent anti-epileptogenic effects.

## Methods

### Animals

C57BL/6J mice were purchased from GemPharmatech (Chengdu, China). Since the differences in seizure severity between males and females has been well documented 24, for all of the *in vivo* studies, only adult male mice (8-12 weeks old, 22-28 g body weight) were used. The mice were housed under a specific-pathogen-free (SPF) facility under a 12-hour light‒dark cycle, with controlled temperature (24±2 °C) and humidity (40-60%). Each cage contained 4-5 animals with *ad libitum* access to standard chow and water. Certified technicians monitored all mice daily for body condition, activity levels and other welfare indicators. For surgical interventions, mice were anesthetized with isoflurane (4-5% induction, 2-3% maintenance in 100% O₂) using a precision vaporizer. Anesthesia depth was verified by absent toe-pinch reflexes and stable respiratory rates. Perioperative analgesia was achieved with carprofen (5 mg/kg, intraperitoneal) administered 1 hour pre-surgery and repeated every 24 hours for 48 hours post-surgery. Postoperative recovery was monitored via body weight and pain-related behaviors (e.g., reduced grooming, mobility). All efforts were maximized to reduce both the suffering and the number of animals. The animal experiments were approved by the Animal Ethics Committee of Southwest Medical University and were conducted in accordance with the National Institutes of Health Guidelines for the Care and Use of Laboratory Animals.

### Chemicals

The following chemicals were used in this study: Kainic acid (KA; MedChemExpress, catalog no. HY-N2309), pentylenetetrazole (PTZ; Sigma‒Aldrich, catalog no. P6500), OICR-9429 (MedChemExpress, catalog no. HY-16993, purity: 99.71%), tetrodotoxin (TTX; Tocris Bioscience, catalog no. 1078), picrotoxin (PTX; Sigma‒Aldrich, catalog no. P1675), D-AP5 (Tocris, catalog no. 0106), NBQX disodium salt (Tocris, catalog no. 1044), and Sulfobutylether-β-Cyclodextrin (SBE-β-CD; MedChemExpress, catalog no. HY-17031). All patch-clamp recording reagents were obtained from Sigma‒Aldrich (St. Louis, MO, USA) unless otherwise noted. For *in vivo* administration, OICR-9429 was first prepared as a 25 mg/mL stock solution in DMSO. Immediately before use, this stock was mixed at a 1:9 ratio with 20% (w/v) SBE-β-CD in saline to yield a 2.5 mg/mL working solution. The working solution was administered via intraperitoneal injection at doses of 2.5, 5, or 10 mg/kg, with the injection volume adjusted according to individual mouse body weight. Control animals received an equivalent volume of the vehicle (a 1:9 mixture of DMSO and 20% SBE-β-CD saline solution), with the volume matched to that used for the 10 mg/kg group.

### Adeno-associated viruse (AAV) production

The preparation of AAV vectors was performed by GeneChem (Shanghai, China). For neuron-specific WDR5 manipulation, we constructed the following AAV9 vectors: (1) knockdown vectors (GV680) featuring hSyn promoter-driven eGFP followed by three distinct WDR5-targeting RNAi sequences (RNAi #1: 5'-CAGTCTCAGCCGTTCATTTCA-3'; RNAi #2: 5'-ATCCTCCAGTGTCCTTCGTGA-3'; RNAi #3: 5'-GTCACACAGATGTTGTGATTT-3') and one scramble control (5'-TTCTCCGAACGTGTCACGT-3') embedded in the MIR155 scaffold, with WPRE enhancement and SV40 polyA termination; and (2) for the most effective RNAi #3, we engineered a synonymous mutation-resistant rescue vector (GV475) expressing full-length mouse Wdr5 (NM_080848.2 with silent mutations in the RNAi-#3 target region, 5'-GcCAtACgGAcGTgGTtATcT-3'), under hSyn promoter control with the C-terminal cherry-3FLAG reporter and SV40 polyA, alongside an empty hSyn-cherry-3FLAG control vector, all packaged as AAV9 with titers >1×10¹³ vg/mL. For neuron-specific ARID5B knockdown, we generated AAV9 vectors (GV680) containing the hSyn promoter-driven eGFP reporter followed by three distinct RNAi sequences (RNAi #1: 5'-CCTTCTTAGTGGCCCTGTATA-3'; RNAi #2: 5'-ATCCGTAATCCAACATGTTCA-3'; RNAi #3: 5'-ATCTCTCCACCTGCAAGATAA-3') embedded in the MIR155 scaffold, with WPRE and SV40 polyA elements, where RNAi #1 demonstrated the most efficient ARID5B knockdown *in vivo*.

### Stereotaxic injection of AAV

The adult mice were anesthetized with isoflurane and secured in a mouse-adapted stereotaxic frame (RWD Life Sciences, Shenzhen, China), with the head maintained in a horizontal plane and the body temperature regulated at 37 °C using a heating pad. Following hair removal and skin disinfection, a 1-cm midline incision was made to expose the bregma and lambda sutures. Hippocampal CA1 coordinates (-2.0 mm anteroposterior (AP), -1.5 mm mediolateral (ML) from bregma, and -1.5 mm dorsoventral (DV) from the dura) were determined according to the Paxinos and Franklin mouse brain atlas 25. After a small craniotomy at the target site was drilled using a microdrill, a total volume of 0.3 μL of AAV (2-4×10¹² vg/mL) was injected into each CA1 region at a rate of 0.05 μL/min using a Hamilton syringe, followed by a 10-minute postinjection needle retention period to minimize backflow before slow withdrawal. Following surgery, AAV-injected mice were housed for postoperative recovery, and body weight and behavioral status were monitored daily. When dual-AAV coinjection was needed, equal titers of each virus (1:1 ratio) were thoroughly mixed in virus dilution buffer (150 mM NaCl, 10 mM Tris-HCl, pH 7.4). The mixed AAVs were then stereotaxically injected into hippocampus. AAV expression verification and any other experiments were performed 3-4 weeks after injection.

### Intrahippocampal KA injection-induced TLE model (IHKA-TLE)

Adult mice were anesthetized with isoflurane and securely mounted in a stereotaxic apparatus with the head positioned symmetrically in the horizontal plane. Following the aforementioned stereotactic injection protocol, KA (100 nL, 20 mM in saline for the IHKA group) or an equivalent volume of saline (for the sham control group) was slowly delivered into the right hippocampal CA1 region over 3 minutes using a Hamilton syringe, with the needle retained for 5 minutes postinjection to prevent reflux before gradual withdrawal. Seizures were scored using a modified Racine scale 26: stage 1, chewing or facial clonus; stage 2, chewing and head nodding or shaking; stage 3, unilateral forelimb clonus; stage 4, bilateral forelimb clonus with rearing; stage 5, bilateral forelimb clonus with rearing and falling; and stage 6, status epilepticus (SE) characterized by continuous epileptiform activity or recurrent seizures (stages 3-5). Consistent with previous studies, mice that successfully developed SE following IHKA injection typically exhibited electrographic and electroclinical SRSs after a short latent period of 2-7 days. In the behavioral studies of this research, the overall mortality rate for IHKA-injected mice was 13.04%. This data includes animals that were euthanized due to respiratory distress during the acute phase, in strict accordance with our ethical protocol. Among all mice that survived after SE, 98.55% successfully developed SRSs. All the mice that reached Racine stage 6 were included in the subsequent experiments. Three hours after SE induction, diazepam (10 mg/kg) was administered to alleviate convulsive seizures. In strict accordance with ethical protocols, mice with labored respiration or respiration arrest were immediately euthanized.

### PTZ kindling

PTZ, a competitive GABA_A_R antagonist, was used to establish the classic acute seizure model 27. Acute seizures were induced by the intraperitoneal injection of freshly prepared PTZ (70 mg/kg in saline). Age- and weight-matched control mice received equivalent saline injections. Seizure activity was continuously monitored for 120 minutes postinjection for behavioral assessment, with Racine stages 4-6 classified as generalized convulsive seizures on the basis of the modified Racine scale (as described above). Three hours after PTZ administration, hippocampal tissues were dissected for western blot (WB) analysis.

### Electrode implantation

The mice were anesthetized with isoflurane and securely positioned in a stereotaxic frame. Four small burr holes were drilled through the skull to the dura mater using a microdrill (0.5 mm diameter, RWD Life Sciences), with two holes positioned bilaterally over the cerebellar cortex for reference and ground electrodes and two holes over the bilateral temporal lobe cortex for recording electrodes. A prefabricated electroencephalography (EEG) head mount was fixed to the skull using four stainless-steel screws threaded into the drilled holes, and silver conductive paint was applied around the screw threads to ensure low-impedance electrical contact with the head mount. Two bipolar electromyography (EMG) electrodes affixed to the EEG head mount assembly were surgically implanted bilaterally into the nuchal musculature to record electromyographic signals, after which the entire implant was securely insulated and stabilized with dental acrylic cement. Postoperatively, the mice were individually housed in standard cages and allowed to recover for a minimum of 7 days with ad libitum access to analgesics and softened food. Mice exhibiting a > 15% reduction in body weight or exhibiting neurological abnormalities during the first 7 days following implantation were excluded from subsequent video-EEG recordings.

### Video-EEG recordings and seizure analysis

For video-EEG recording, the head-mounted electrode assembly was interfaced with the amplification and signal acquisition system using flexible, low-noise shielded cables (Pinnacle Technology, USA). Each mouse was individually housed in a transparent plexiglass recording chamber (30 × 30 × 40 cm) with ad libitum access to standard rodent chow and water while unrestricted ambulatory activity was maintained. EEG signals were continuously acquired at a sampling frequency of 1 kHz using a synchronized video-EEG monitoring system (Pinnacle Technology), with signals bandpass-filtered between 1-100 Hz. Spontaneous recurrent seizures (SRSs) were analyzed using Sirenia Seizure Pro software (Pinnacle Technology). All the EEG traces were manually scrutinized for SRS occurrence, operationally defined as (1) abrupt onset of high-amplitude (> 2× baseline) rhythmic discharges and (2) a minimum duration of 10 seconds, which is consistent with established criteria 28. Isolated spike bursts or periodic interictal spikes were excluded from SRS quantification. All EEG-identified SRS events were temporally correlated with synchronized video recordings for behavioral phenotyping, with a particular focus on Racine stage 4-6 seizures. The daily frequency of SRS was determined by normalizing the total SRS count to a 24-hour period on the basis of the actual recording duration. All analyses were conducted by experimenters who were blinded to the treatment group allocation.

### Primary hippocampal neuron culture

Primary hippocampal neurons were isolated from postnatal day 0-1 (P0-P1) C57BL/6J littermates using an established protocol with modifications. Briefly, neonatal pups were euthanized by cervical dislocation, followed by rapid brain extraction and hippocampal dissection. The isolated tissues were immediately immersed in ice-cold Ca²⁺/Mg²⁺-free Hank's balanced salt solution (HBSS; Gibco, catalog no. 14175095). After enzymatic digestion with 1.25% trypsin (Gibco, catalog no. 25200072) at 37 °C for 10 min, the reaction was terminated by adding HBSS containing 20% fetal bovine serum (FBS; Sigma-Aldrich, catalog no. F8318). Tissue dissociation was achieved through mechanical trituration. The dissociated neuronal suspension was seeded onto poly-L-lysine (0.1 mg/mL; Sigma-Aldrich, catalog no. P4832)-coated 6-well plates (with or without 14 mm coverslips) in plating medium consisting of Dulbecco's modified eagle medium (DMEM) supplemented with 10% FBS and 1% penicillin-streptomycin (Gibco, catalog no. 15140122). Four hours after plating, the plating medium was completely replaced with neuronal culture medium consisting of neurobasal medium (Gibco, catalog no. 21103049) supplemented with 2% B-27 supplement (Gibco, catalog no. 17504044), 1% L-glutamine (Gibco, catalog no. A2916801), and 1% penicillin-streptomycin. Beginning on the following day (day *in vitro* 1, DIV1), half of the medium was replaced every 2-3 days with fresh culture medium. The cells were maintained at 37 °C in a 5% CO₂ humidified incubator. To establish the *in vitro* KA model, cultures were pretreated with 5 μM KA added to the culture medium for 24 hours prior to the subsequent assays. To achieve AAV transduction, primary neurons at 2-3 DIV were exposed to AAV at a multiplicity of infection (MOI) of 1×10⁴-1×10⁵. After 24 h of incubation, half of the culture medium was replaced with fresh medium. Neurons were maintained until DIV14 before experimental assays.

### Total protein extraction

For hippocampal tissue protein extraction, RIPA lysis buffer (Beyotime Biotechnology, catalog no. P0013B) was prepared by adding phenylmethanesulfonyl fluoride (PMSF; Beyotime Biotechnology, catalog no. ST506) from a 100 mM stock solution to achieve a final concentration of 1 mM immediately before use. Following the decapitation of deeply anesthetized mice, hippocampal tissues were rapidly dissected and homogenized in ice-cold RIPA buffer (100 μL per 10 mg of tissue) using a mechanical homogenizer. The lysates were centrifuged at 12,000 × g for 15 min at 4 °C, after which the supernatants were collected for protein quantification, normalized, denatured by boiling (95-100 °C, 5 min), and stored at -80 °C.

For primary neuronal cultures, the medium was aspirated, and the cells were washed once with ice-cold PBS before lysis in RIPA buffer (100 μL per well of a 6-well plate). After complete lysis, the samples were centrifuged (12,000 × g, 15 min, 4 °C), and the supernatants were processed identically for quantification, normalization, heat denaturation, and storage at -80 °C.

### Nuclear protein extraction

Nuclear proteins were isolated from hippocampal tissues using a Minute™ Cytosolic and Nuclear Extraction Kit for Frozen/Fresh Tissues (Invent Biotechnologies, catalog no. NT-032) following the manufacturer's protocol. Briefly, protease inhibitors were added to an appropriate volume of Buffer A (provided in the kit) immediately before use. After decapitation of the deeply anesthetized mice, the hippocampi were dissected and homogenized in 250 μL of ice-cold Buffer A, followed by 5 min of incubation on ice and centrifugation at 14,000 × g for 5 min. The supernatant was discarded, and the pellet was resuspended in 0.8 mL of Buffer A with additional homogenization. Following 8 min of incubation on ice, 0.7 mL of the supernatant was transferred to a new 1.5 mL tube and centrifuged at 500 × g for 2 min. After removing the supernatant, the pellet was treated with 0.5 mL of Buffer B for 5 min on ice and then centrifuged at 2,000 × g for 2 min. The nuclear fraction was finally resuspended in 50 μL of Buffer D supplemented with 100 μL of Buffer A, homogenized, and clarified by centrifugation (14,000 × g, 5 min), with the resulting supernatant containing the extracted nuclear proteins.

### Membrane protein extraction

Membrane proteins were isolated from hippocampal tissues using a Minute™ Plasma Membrane/Protein Isolation and Cell Fractionation Kit (Invent Biotechnologies, catalog no. SM-005) according to the manufacturer's instructions. Briefly, hippocampi were dissected from deeply anesthetized mice following decapitation and homogenized in 200 μL of Buffer A for 1 min, followed by the addition of 300 μL of Buffer A and incubation on ice for 5 min. After centrifugation, the supernatant was transferred to a new 1.5 mL tube and centrifuged at 16,000 × g for 30 min at 4 °C. The pellet was resuspended in 200 μL of Buffer B and centrifuged at 7,800 × g for 5 min at 4 °C. The resulting supernatant was carefully transferred to a new 2.0 mL tube, mixed with 1.6 mL of ice-cold PBS, and centrifuged at 16,000 × g for 30 min. The final pellet containing the plasma membrane fraction was dissolved in 100 μL of Minute™ Denaturing Protein Solubilization Reagent (Invent Biotechnologies, WA-009) and stored for subsequent analysis.

### WB analysis

SDS‒polyacrylamide gels were prepared at concentrations optimized for the molecular weights of the target proteins. Following electrophoretic separation, the proteins were transferred onto polyvinylidene difluoride (PVDF) membranes (Merck Millipore) using standard wet transfer protocols. The membranes were blocked with 5% nonfat dry milk in TBST (Tris-buffered saline with 0.1% Tween-20) for 1 h at room temperature (RT) before being incubated with primary antibodies (manufacturer-recommended dilutions) overnight at 4 °C. After three 10-min TBST washes, the membranes were probed with horseradish peroxidase (HRP)-conjugated secondary antibodies (1:5,000 dilution; RT, 1 h). Following additional TBST washes (3 × 10 min), immunoreactive bands were visualized using enhanced chemiluminescence (ECL) substrate and imaged with a ChemiDoc imaging system (Bio-Rad). Densitometric analysis was performed using ImageJ software (NIH, USA). For sequential detection of multiple proteins, the membranes were stripped with Western Stripping Buffer (Weakly Alkaline) (Beyotime, catalog no. P0025B) at room temperature for 10 minutes and subsequently reprobed with the respective primary antibodies. This buffer is designed to remove primary and secondary antibodies by disrupting non-covalent bonds under mild, weakly alkaline conditions with minimal impact on the target antigens. Signal intensities were quantified for all samples following background subtraction, with normalized values calculated relative to corresponding internal reference proteins. Complete antibody information is provided in Table S1.

### Co-immunoprecipitation (Co-IP)

Protein complexes were immunoprecipitated using protein A/G magnetic beads (MedChemExpress, catalog no. HY-K0202) in combination with cell lysis buffer for WB analysis/IP (Beyotime, catalog no. P0013), and all procedures were performed according to the manufacturers' optimized protocols. Briefly, protein lysates were extracted using cell lysis buffer for WB analysis/IP and maintained on ice. For each reaction, 40 μL of magnetic beads were washed three times with 400 μL of PBST (1× PBS containing 0.5% Tween-20, pH 7.4) in a 1.5 mL microcentrifuge tube. The WDR5 antibody was diluted to a final concentration of 10 μg/mL in 400 μL of PBST and incubated with the prewashed beads on a rotary mixer at 4 °C for 2 h. After magnetic separation, the bead‒antibody complexes were collected and washed four times with PBST. Subsequently, 50 μL of 1× SDS-PAGE loading buffer was added to the beads, followed by thorough mixing and denaturation at 95 °C for 5 min. The supernatant containing the eluted proteins was magnetically separated and subjected to WB analysis. Complete antibody information is provided in Table S1.

### Immunofluorescence

Following deep anesthesia, the mice were transcardially perfused with ice-cold PBS followed by 4% paraformaldehyde (PFA). The brains were dissected and postfixed overnight in 4% PFA at 4 °C and then cryoprotected in 30% sucrose/PBS at 4 °C until saturation. Using a cryostat (Leica Biosystems, CM1850), 16-µm-thick coronal sections spanning the dorsal-ventral hippocampal axis were prepared and placed on slides. For immunostaining, the sections were permeabilized with 0.4% Triton X-100/PBS (Sigma‒Aldrich) and then blocked with 5% normal goat serum (NGS) for 1 h at RT. Primary antibody incubation was performed at 4°C overnight using the manufacturer-recommended dilutions in PBS. After the samples were washed with PBS (3×5 min), species-matched Alexa Fluor-conjugated secondary antibodies were applied for 1 h at RT. After staining, Fluoromount-G with DAPI (SouthernBiotech, catalog no. 0100-20) was added to the slices, and the coverslips were mounted. Complete antibody information is provided in Table S1.

For validation of AAV transduction efficiency, primary hippocampal neurons transfected with AAV at DIV3 and cultured on coverslips in 6-well plates until DIV14 were processed as follows: after a single PBS wash, the cells were fixed with 4% PFA/4% sucrose in PBS for 20 min at room temperature, followed by three 5-min washes with PBS and blocking with 5% NGS for 1 h at RT. The neurons were then incubated overnight at 4°C with primary antibodies against eGFP and microtubule-associated protein 2 (MAP2) diluted in PBS containing 3% NGS and 0.2% Triton X-100, washed three times with PBS (5 min each), and incubated with species-matched secondary antibodies for 1 h at RT before being washed with PBS and mounted with Fluoromount-G (SouthernBiotech, catalog no. 0100-01). For surface GABA_A_R labeling, neurons were transfected with either shScr or shWDR5 at DIV3, maintained until DIV14, and treated with 10 μM KA at DIV14 before immunostaining at 24 h posttreatment. Live DIV15 neurons were incubated with extracellular domain-targeting primary antibodies against GABA_A_R α1 (Sα1) or α2 (Sα2) subunits diluted in neuronal culture medium in an incubator with 5% CO2 for 30 min at 37 °C, followed by fixation with 2% PFA/4% sucrose for 15 min and processing using the aforementioned immunostaining protocol without permeabilization. Complete antibody information is provided in Table S1.

Fluorescence images of brain sections and primary neurons were acquired on a Zeiss LSM 980 confocal microscope, with consistent acquisition parameters (laser power, gain, offset, and pinhole size) maintained throughout all comparable experimental groups. Subsequent image processing and fluorescence intensity quantification were performed using ZEN 2.3 software (Carl Zeiss) and FIJI/ImageJ (National Institutes of Health).

### RNA extraction and quantitative real-time PCR (qPCR)

Total RNA was isolated from hippocampal tissues using an RNApre Pure Tissue Kit (TIANGEN, catalog no. DP431) following the manufacturer's protocol. The RNA purity and concentration were assessed spectrophotometrically (NanoDrop Technologies, Thermo Scientific, USA), with 260/280 nm absorbance ratios consistently ranging between 1.8 and 2.0. cDNA synthesis was performed with HiScript II Q RT SuperMix for qPCR (+gDNA wiper) (Vazyme, catalog no. R223-01) to remove genomic DNA contamination and generate first-strand cDNA. qPCR assays were conducted using ChamQ Universal SYBR qPCR Master Mix (Vazyme, catalog no. Q711) on a Thermo Fisher Scientific real-time PCR system. Gene-specific primer pairs (Table S2) were designed via Primer-BLAST (NCBI) to ensure target specificity. All reactions were performed in technical triplicate. Transcript levels were normalized to the endogenous reference gene *Actb*, and relative gene expression was quantified using the comparative ΔΔCT method (2^-ΔΔCT^).

### Chromatin immunoprecipitation sequencing (ChIP-seq) and data analysis

Two days after surgery, hippocampal tissues ipsilateral to the injection site were collected from the mice in the IHKA and sham groups for ChIP-seq, which was performed by LC-Bio Technology Co., Ltd. (Hangzhou, China). The experimental procedures were as follows: DNA‒protein crosslinking was performed by treating cells with 1% formaldehyde, followed by quenching with 125 mM glycine. Chromatin was extracted using lysis buffer and sonicated to generate DNA fragments ranging from 100 to 500 bp in length. Immunoprecipitation was carried out by incubating chromatin with H3K4me3 antibody-coated bead complexes overnight at 4 °C. Sequencing libraries were prepared from the immunoprecipitated DNA using the NEBNext Ultra™ DNA Library Prep Kit for Illumina (NEB, catalog no. E7645L) with multiplexing indices. Library fragments were size-selected and purified using AMPure XP beads. Quantification was performed using a Qubit fluorometer, and the insert size distribution was assessed using a high-sensitivity DNA chip. Finally, paired-end sequencing (150 bp) was conducted on an Illumina NovaSeq 6000 platform. Raw data in FASTQ format were initially processed using fastp (v0.20.1) 29 to obtain high-quality clean reads by removing adapter sequences, poly-N-containing reads, and low-quality reads. The clean reads were then aligned to the reference genome using Bowtie2 (v2.4.2) 30. Peak calling was performed using MACS2 (v2.2.7.1) 31 with default parameters, and the resulting peaks were visualized using IGV 32. Peak annotation was conducted using the ChIPseeker 33 R package. For motif analysis, both de novo and known motifs were identified using HOMER. Differential peak analysis was performed using either the MAnorm R package (for unreplicated experimental designs) or the DiffBind R package (for replicated experimental designs). Complete antibody information is provided in Table S1.

### RNA sequencing (RNA-seq) and data analysis

Hippocampal tissues ipsilateral to the injection site were collected from deeply anesthetized IHKA-2d mice treated with or without OICR-9429 (10 mg/kg, intraperitoneal injection) for RNA sequencing performed by LC-Bio Technology Co., Ltd. Total RNA was isolated using TRIzol reagent (Thermo Fisher, catalog no. 15596018), followed by quality control assessment of RNA quantity and purity using a NanoDrop ND-1000 instrument and integrity verification. PolyA-containing mRNA was specifically enriched through two rounds of oligo(dT) bead purification. The purified mRNA was fragmented using magnesium-based fragmentation reagents at elevated temperatures, followed by first-strand cDNA synthesis using reverse transcriptase. Second-strand cDNA was synthesized using E. coli DNA polymerase I and RNase H, and dUTP solution (Thermo Fisher, catalog no. R0133) was used to generate double-stranded DNA with blunt ends. After adenylation of the 3' ends, adapters with T-overhangs were ligated, followed by size selection (300±50 bp) using magnetic beads to construct strand-specific libraries. Sequencing was performed on an Illumina NovaSeq 6000 platform to generate 150 bp paired-end reads. The raw sequencing data were initially processed to obtain high-quality clean reads by removing adapter sequences and low-quality bases. The clean reads were then aligned to the reference genome for subsequent analyses including gene expression quantification, GSEA, differential gene expression analysis, and functional enrichment analysis.

### ChIP‒qPCR

ChIP‒qPCR was performed using an EpiQuik™ Tissue Chromatin Immunoprecipitation Kit (EpigenTek, catalog no. P-2003) following the manufacturer's protocol. Briefly, the ChIP assay was performed using strip wells precoated with antibodies (2‒3 µg of specific antibody or 1 µl of nonimmune IgG control per well) in CP2 buffer, followed by 60‒90 min of incubation at RT. Hippocampal tissue samples were dissociated, crosslinked with 1% formaldehyde for 15‒20 min, quenched with glycine, and homogenized in ice-cold buffer. Cell lysates were prepared in CP3 buffer with protease inhibitors and sonicated (4‒5 pulses of 15‒20 sec at power level 2) to shear the DNA. The cleared lysates were diluted 1:1 with CP4 buffer and immunoprecipitated for 60‒90 min at 22‒25 °C. After extensive washing, protein‒DNA complexes were eluted and reverse crosslinked at 65 °C in CP5/CP6 buffers containing proteinase K. DNA was purified using spin columns with sequential ethanol washes (70% and 90%) and eluted in CP8 buffer. The input DNA controls were processed in parallel throughout the procedure. The DNA was stored at -20 °C for subsequent qPCR analysis. Fold enrichment was calculated as 2^(Ct[IgG] - Ct[Specific Antibody]), with input DNA (2% of total chromatin) serving as the normalization control. All experiments included technical triplicates. Antibody and primer information is provided in Table S2.

### Acute brain slice preparation

Following anesthesia, the mice were decapitated, and their brains were rapidly extracted and immersed in ice-cold high-sucrose cutting solution (in mM: 220 sucrose, 26 NaHCO₃, 10 D-glucose, 2.5 KCl, 1.25 NaH₂PO₄, 6 MgCl₂, and 1 CaCl₂, pH 7.3‒7.4). Coronal hippocampal slices (300-µm-thick) were prepared using a vibratome (Leica VT1200S, Germany) and were maintained in the cutting solution. Slices were subsequently transferred to a recovery chamber containing standard artificial cerebrospinal fluid (aCSF; composition in mM: 124 NaCl, 3 KCl, 1.25 NaH₂PO₄, 26 NaHCO₃, 10 glucose, 2 MgCl₂, and 2 CaCl₂, pH 7.3-7.4, 290-300 mOsm) at 32 °C for 30 min, followed by equilibration at RT (25 °C) for ≥ 30 min before experimentation. All the solutions were continuously bubbled with 95% O_2_ and 5% CO_2_.

### Whole-cell patch-clamp recordings

All electrophysiological recordings were conducted in the CA1 region of hippocampal slices maintained in a perfusion chamber mounted on a Nikon E600FN upright microscope equipped with both infrared differential interference contrast (IR-DIC) and epifluorescence optics. Patch-clamp recordings were obtained at RT with borosilicate pipettes (3-6 MΩ; Sutter Instrument) filled with the appropriate intracellular solution (0.22 μm filtered), while oxygenated aCSF (95% O₂/5% CO₂) was continuously perfused (2-3 mL/min) using a peristaltic pump (WPI). Fluorescent CA1 pyramidal neurons were identified on the basis of eGFP/mCherry expression and characteristic morphological features. Recordings were acquired using a Multiclamp 700B amplifier (Molecular Devices) interfaced with Clampex 10.7 software (Molecular Devices), with signals low-pass filtered at 2 kHz and sampled at 10 kHz. Neurons were excluded from analysis if they exhibited unstable resting membrane potential (RMP; fluctuation > 5 mV/min) or depolarized RMP (≤ -50 mV), series resistance > 25 MΩ, or > 20% change in series resistance during recording.

For current-clamp recordings, the intracellular solution contained (in mM): 130 K-methanesulfonate, 10 HEPES, 5 Na-phosphocreatine, 5 KCl, 2 MgCl₂, 0.6 EGTA, 2 Mg-ATP, and 0.3 Na-GTP (pH 7.2-7.3, 285-300 mOsm). Synaptic transmission was blocked by adding the following antagonists to the aCSF: 50 μM D-APV (NMDAR antagonist), 10 μM NBQX (AMPAR antagonist), and 100 μM picrotoxin (GABA_A_R antagonist), thereby eliminating network-driven activity. A series of depolarizing current steps (500 ms duration, -100 to +200 pA range, 20 pA increments) were applied to assess neuronal excitability. The RMP was recorded immediately upon establishing the whole-cell configuration in current-clamp mode (I = 0). The input resistance (R_in_) was calculated using Ohm's law (Rin = ΔV/ΔI) from the voltage response to a 20-pA current injection. The rheobase was determined as the minimal 500-ms current pulse required to elicit at least one AP. AP properties were analyzed from averaged traces generated by suprathreshold stimulation (rheobase + 20 pA). The AP threshold was defined as the membrane potential corresponding to the point where the first temporal derivative (dV/dt) reached 10% of its maximum value during the AP rising phase. The AP amplitude was quantified from the resting potential to peak depolarization. The medium afterhyperpolarization (mAHP) amplitude was measured as the maximal hyperpolarization relative to the prespike baseline following a spike train.

For miniature and evoked EPSC recordings, neurons were voltage-clamped at -70 mV in oxygenated aCSF supplemented with 100 µM PTX, while the intracellular solution contained the following (in mM): 130 Cs-methanesulfonate, 10 HEPES, 10 CsCl, 4 NaCl, 1 MgCl₂, 1 EGTA, 5 N-methyl-D-glucamine (NMG), 5 Mg-ATP, 0.5 Na₂-GTP, and 12 phosphocreatine (pH 7.2-7.3, 275-290 mOsm). IPSCs were recorded using aCSF supplemented with 10 μM NBQX and 50 μM D-AP5 to block glutamatergic transmission, with neurons voltage clamped at -70 mV for miniature IPSCs (mIPSCs) and at 0 mV for evoked disynaptic IPSCs, using a high-Cl⁻ intracellular solution (in mM: 10 HEPES, 30 NMG, 100 CsCl, 1 MgCl₂, 12 phosphocreatine, 0.5 Na₂-GTP, 5 Mg-ATP, and 1 EGTA; pH 7.2-7.3, 275-290 mOsm). TTX (1 µM) was included in aCSF for all miniature current recordings (mEPSCs/mIPSCs). Synaptic responses were evoked by stimulating Schaffer collateral fibers using concentric bipolar electrodes. The stimulation intensity was adjusted to elicit approximately 50% of the maximal response amplitude. The paired-pulse ratio (PPR) was calculated as P2/P1 from ≥ 10 averaged traces, with the paired stimuli delivered at 50-ms interstimulus intervals. For excitatory/inhibitory (E/I) current ratios, EPSCs (-70 mV) and IPSCs (0 mV) were sequentially recorded in the same neuron, with the ratio derived from averaged peak amplitudes (10 traces each). For evoked IPSC recordings, hippocampal CA1 pyramidal neurons were stimulated at Schaffer collaterals with incrementing intensities (0-200 μA in 50-μA steps). To confirm the GABA_A_R-mediated nature of the recorded currents, picrotoxin (PTX; 100 μM) was bath-treated for 20 minutes after the selected experiments. All electrophysiological data were analyzed offline using either MiniAnalysis software (v6.0.3; Synaptosoft, Leonia, NJ) or Clampfit (Molecular Devices). For quantitative analysis of miniature synaptic events, continuous 180-second recordings were acquired.

### Statistical analysis

Statistical analyses were performed using GraphPad Prism 8 (GraphPad software, USA). Sample sizes were determined on the basis of prior experience in calculating experimental variability. Statistical parameters, significance, and the exact n values are reported in the figure legends and statistical source data. Before running the statistical tests, the normality and variance of the distributions were determined. Two-tailed unpaired Student's t-test was used to compare two groups. For multigroup comparisons, one-way ANOVA or two-way ANOVA was used. All post-hoc tests accounted for multiple comparisons. Intergroup differences in mortality were analyzed by a chi-square test. Survival rates were analyzed using Kaplan‒Meier curves, and intergroup differences were assessed by the log-rank test. The quantitative data are presented as the means ± standard deviations (SDs). The significance level was set at p < 0.05.

## Results

### H3K4me3 increase and remodeling accompany epileptogenesis

**Figure 1:**
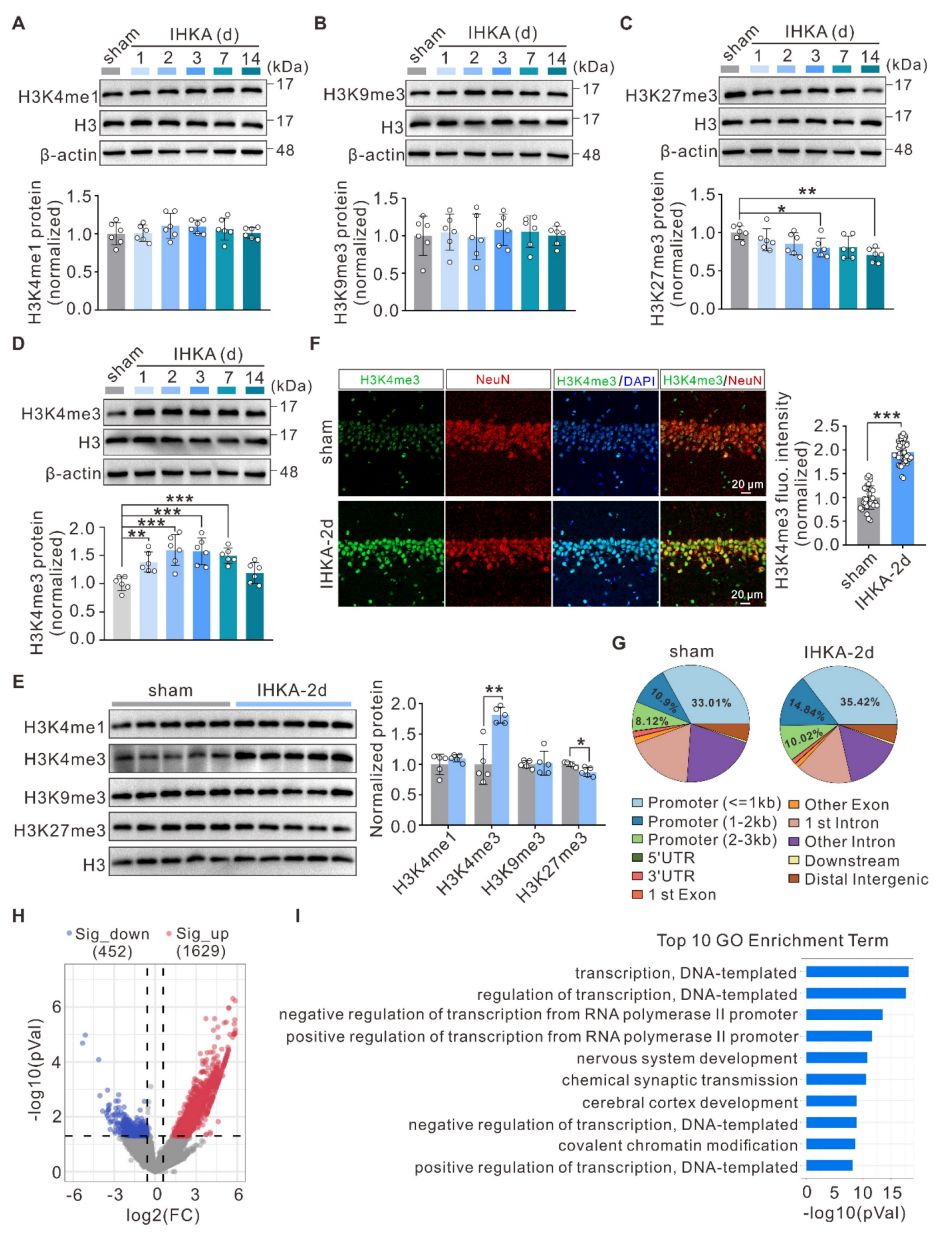
** H3K4me3 upregulation and genomic repositioning in epileptogenesis. (A-D)** Representative immunoblots (top) and quantitative analysis (bottom) of H3K4me1 (**A**), H3K9me3 (**B**), H3K27me3 (**C**), and H3K4me3 (**D**) in the ipsilateral hippocampus following IHKA microinjection. β-actin was used as a loading control. n = 6 biologically independent mice per group. **(E)** Representative immunoblots (left) and quantitative analysis (right) of nuclear H3K4me1, H3K4me3, H3K9me3, and H3K27me3 in the ipsilateral hippocampus 2 days after IHKA microinjection. Histone H3 was used as a loading control. n = 5 biologically independent mice per group. **(F)** Left: Representative confocal images of the hippocampal CA1 from sham control and 2 d post-IHKA mice, stained for H3K4me3 (green), neuronal marker neuron-specific nuclear protein (NeuN, red), and 4',6-diamidino-2-phenylindole (DAPI, blue), and presenting the H3K4me3 single channel, NeuN single channel, and merged H3K4me3/DAPI and H3K4me3/NeuN channels, respectively. Right: Quantification of H3K4me3 mean fluorescence intensity. n = 32 fields (sham, 4 mice), n = 40 fields (IHKA-2d, 5 mice). **(G)** Genomic distribution of H3K4me3 peaks in sham and 2 d post-IHKA hippocampal tissues. Pie charts show the proportion of peaks annotated to promoters, 5′UTRs, exons, introns, 3′UTRs, and intergenic regions. Peak calling was performed using MACS2 (q < 0.01) with RefSeq gene annotations. n = 3 biologically independent ChIP-seq experiments. **(H)** Volcano plot of genes with differential H3K4me3 peak enrichment (Fold Change > 1.5, P < 0.05) in hippocampal tissues 2 d post-IHKA versus sham control. **(I)** Gene Ontology biological process (GO-BP) enrichment analysis for genes showing changed H3K4me3 occupancy in (h). Top 10 significant terms (FDR < 0.05) are shown. *p < 0.05, **p < 0.01, ***p < 0.001. Statistical analyses were performed as follows: one-way ANOVA with Dunnett's post hoc test (Figure 1A-D); two-tailed unpaired Student's t-tests with Holm-Šidák correction for multiple comparisons (Figure 1E); two-tailed unpaired Student's t-tests (Figure 1F).

To investigate the role of histone methylation in epileptogenesis, we employed the intrahippocampal kainic acid-induced TLE (IHKA-TLE) mouse model, which is a well-established model that closely recapitulates many of the pathological and clinical features observed in human TLE 34. Notably, almost all the mice that received the IHKA injections and successfully developed status epilepticus (SE) subsequently exhibited spontaneous recurrent seizures (SRSs) following a short latency period of 2-7 days 35. Considering that the hippocampus serves as a key driver in TLE 36, we first evaluated the expression profiles of several of the most common histone methylation marks in the ipsilateral hippocampus of IHKA-TLE model mice. Western blot (WB) analysis revealed that the total protein levels of H3K4me1 and H3K9me3 did not significantly change during epileptogenesis (Figure 1A, B). In contrast, the level of H3K27me3 was significantly decreased at 3 and 14 days following IHKA injection (Figure 1C), whereas H3K4me3 expression was significantly increased at 1, 2, 3, and 7 days after IHKA administration (Figure 1D). This dynamic upregulation of H3K4me3 was not observed in the sham control group (Figure S1A). On the basis of its dynamic upregulation across multiple time points during the early epileptogenic phase, we propose that H3K4me3 may functionally participate in epileptogenesis. For subsequent experiments, we selected the 2-day post-IHKA (IHKA-2d) time point as our primary focus, as this stage not only exhibited the most significant increase in H3K4me3 expression but also allowed for experimental analysis during the early phase of epileptogenesis, thereby minimizing secondary interference from neuronal loss and other delayed pathological changes. Consistent with the alterations in total protein levels, the nuclear protein levels of H3K4me3 were significantly increased compared with those in the control group (Figure 1E). We next sought to determine the spatial and cellular distribution of this epigenetic mark. Immunofluorescence staining revealed that under basal conditions, H3K4me3 was primarily distributed in the dentate gyrus (DG) and CA1. Following IHKA induction, a robust enrichment occurred almost exclusively within the CA1 subfield (Figure S1B), establishing this region as the primary site of the observed change. To identify the specific cell types involved, we performed co-localization studies within the CA1 region. The results demonstrated that the seizure-induced H3K4me3 signal was predominantly localized to neuronal nuclei, as evidenced by its pronounced intensity within the CA1 pyramidal cell layer (Figure 1F). In contrast, only relatively weak expression was detected in astrocytes and microglia (Figure S1C, D). Collectively, these findings suggest that the active transcriptional mark H3K4me3 may contribute to epileptogenesis primarily through the modulation of gene expression in hippocampal neurons, with a marked focus in the vulnerable CA1 subfield.

To gain deeper insight into the genome-wide alterations in H3K4me3, we performed chromatin immunoprecipitation sequencing (ChIP-seq) to profile the differential genomic distribution of H3K4me3 in hippocampal tissues between sham control and IHKA-2d mice. The results revealed increased H3K4me3 occupancy at gene promoter regions in IHKA-2d hippocampal tissues compared with that in sham control hippocampal tissues (Figure 1G; Figure S1E). Peak calling analysis revealed 2,081 genomic loci with significant H3K4me3 peak alterations in the hippocampal tissues of IHKA-2d mice compared with sham control mice, comprising 1,629 loci with increased H3K4me3 occupancy and 452 loci with decreased H3K4me3 occupancy (Figure 1H). Gene Ontology (GO) enrichment analysis of genes with different H3K4me3 peaks revealed that the most significantly enriched biological process terms were associated with transcriptional regulation and chemical synaptic transmission (Figure 1I). Kyoto Encyclopedia of Genes and Genomes (KEGG) pathway analysis revealed that the most enriched pathways included the MAPK signaling pathway and pathways associated with glutamatergic synapses, both of which are directly associated with epilepsy (Figure S1F). Collectively, these results demonstrate a global increase in and redistribution of H3K4me3 modifications in the hippocampal genome of IHKA model mice, suggesting that H3K4me3 plays a role in epileptogenesis.

### Pharmacological inhibition of the WDR5-KMT2 complex exerts antiepileptic effects

**Figure 2:**
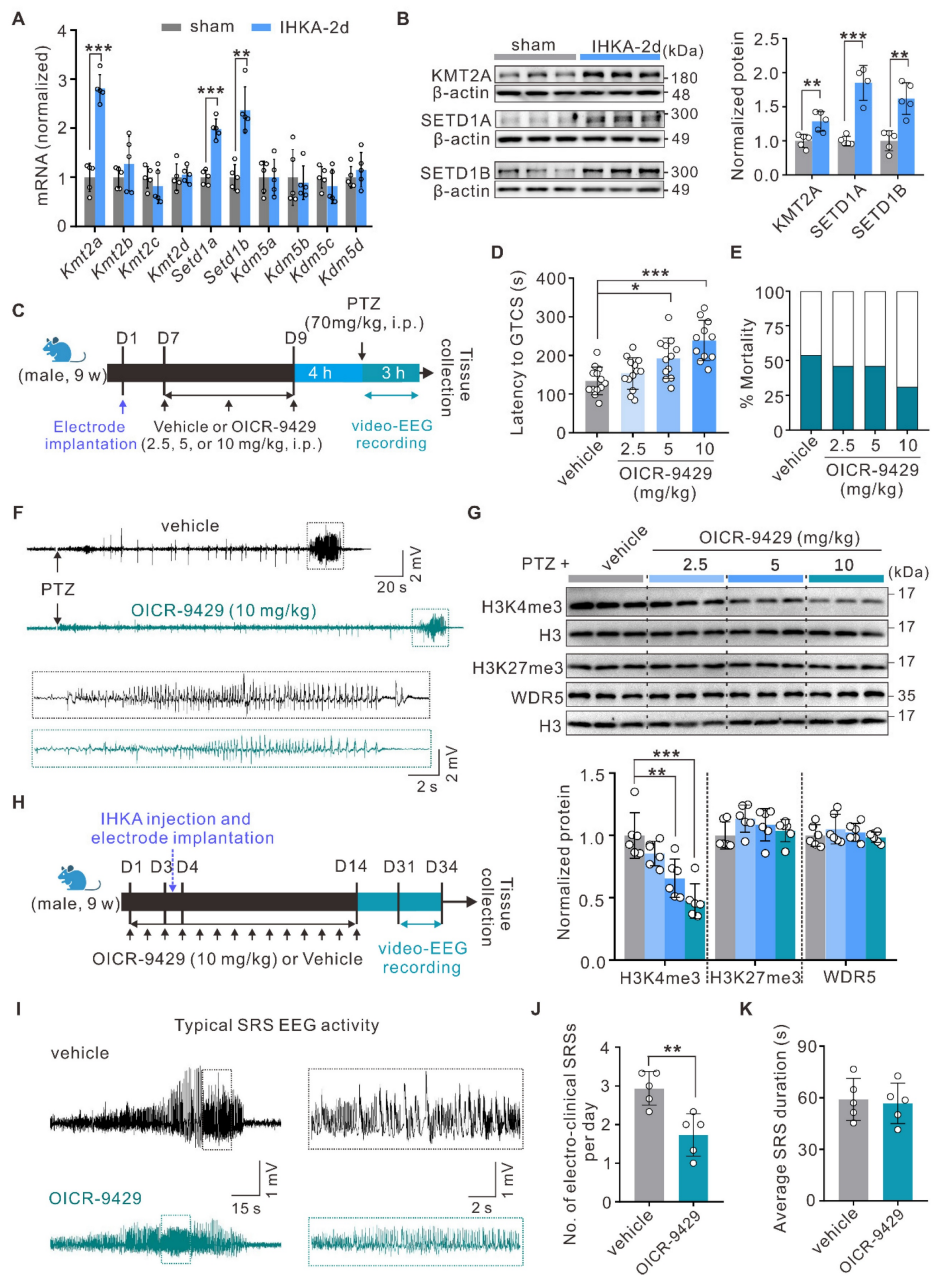
** Inhibition of WDR5-KMT2 complex reduces seizure susceptibility and severity. (A)** qPCR analysis of indicated mRNA expression levels in ipsilateral hippocampal tissues from sham control versus IHKA-2d mice. n = 5 biologically independent mice per group. **(B)** Representative immunoblots (top) and quantitative analysis (bottom) of KMT2A, SETD1A, and SETD1B protein expression in ipsilateral hippocampal tissues from sham control versus IHKA-2d mice. n = 4-5 biologically independent mice per group. **(C)** Schematic diagram of experimental design and timeline. (**D-E**) Pharmacological effects of OICR-9429 (2.5, 5, 10 mg/kg, i.p.) versus vehicle control on latency to GTCS (**D**) and mortality rates (**E**) in the PTZ-induced acute seizure model. n = 13 biologically independent mice per group. **(F)** Representative EEG traces showing seizure activity in PTZ-injected mice pretreated with either vehicle or OICR-9429 (10 mg/kg). The boxed region indicates the magnified epileptiform discharges. **(G)** Representative immunoblots (top) and quantitative analysis (bottom) of nuclear H3K4me3, H3K27me3, and WDR5 protein levels in hippocampal lysates from PTZ-kindled mice pretreated with OICR-9429 (0, 2.5, 5, 10 mg/kg). n = 6 biologically independent mice per group. **(H)** Schematic diagram of experimental design and timeline. **(I)** Representative EEG traces showing typical SRS electrographic activity recorded from chronic IHKA-TLE mice treated with either vehicle or OICR-9429 (10 mg/kg). The boxed region indicates the magnified epileptiform discharges.** (J-K)** Bar graph denotes electroclinical SRS (**J**) daily frequency (SRSs/day) and (**K**) mean duration in chronic IHKA-TLE mice treated with vehicle or OICR-9429 (10 mg/kg, i.p.). n = 5 biologically independent mice per group. No mortality occurred in any group during the behavioral observation period following SE induction. *p < 0.05, **p < 0.01, ***p < 0.001. Statistical analyses were performed as follows: two-tailed unpaired Student's t-tests with Holm-Šidák correction for multiple comparisons (Figure 2A-B); one-way ANOVA with Tukey's post hoc test (Figure 2D); two-tailed unpaired Student's t-tests (Figure 2J-K). In Figure 2G, H3K27me3 protein levels were analyzed using the Kruskal-Wallis test, while other data were assessed with one-way ANOVA with Tukey's post hoc test.

H3K4me3 levels are dynamically regulated by both methyltransferases and demethylases 13. To investigate the potential mechanisms underlying increased H3K4me3 levels in the hippocampi of epileptic mice, we quantified the mRNA expression of methyltransferases (*Kmt2a-d*, *Setd1a-b*) and demethylases (*Kdm5a-d*) using quantitative real-time PCR (qPCR). Compared with sham control mice, IHKA-2d mice presented significantly increased mRNA levels of *Kmt2a*,* Setd1a*, and *Setd1b* in hippocampal tissues (Figure 2A). WB analysis further confirmed the upregulation of KMT2A, SETD1A, and SETD1B at the protein level (Figure 2B). These findings suggest that the observed increase in H3K4me3 levels likely results from coordinated regulation by multiple KMT2 family methyltransferases.

To circumvent potential compensatory effects from targeting individual KMT2 members, we chose to pharmacologically inhibit WDR5-KMT2 complex activity, which is essential for the function of all KMT2 family proteins, to elucidate the functional role of increased H3K4me3 modification in epileptogenesis. As a well-characterized small-molecule inhibitor, OICR-9429 specifically binds to the WDR5 WIN site (WDR5-interaction motif), effectively blocking its association with KMT2 family proteins and consequently suppressing H3K4 methylation activity 37, 38. Following the intraperitoneal administration of OICR-9429 (2.5, 5, or 10 mg/kg) or vehicle control, we performed video-electroencephalography (video-EEG) monitoring in the PTZ acute kindling model, which is an established gold standard for primary screening of antiepileptic compounds due to its high predictive validity 39 (Figure 2C). Behavioral analysis revealed that OICR-9429 treatment significantly prolonged the latency to the first generalized tonic‒clonic seizure (GTCS) in a dose-dependent manner, with a nonsignificant trend toward reduced mortality (Figure 2D-F). To further characterize the molecular signatures in hippocampal tissues following OICR-9429 treatment, we performed WB analysis of nuclear protein extracts after behavioral assessment. The results showed that OICR-9429 selectively reduced hippocampal H3K4me3 but not H3K27me3 levels in a dose-dependent manner (Figure 2G), revealing its abilities to penetrate the brain-blood barrier and specifically target the WDR5-KMT2 complex. Notably, OICR-9429 administration did not alter WDR5 protein expression (Figure 2G). On the basis of these findings, we selected 10 mg/kg OICR-9429 for subsequent experiments. To this end, we confirmed that this regimen significantly reduced global H3K4me3 levels in the hippocampus of normal mice (Figure S2I), thereby verifying successful target engagement in the brain. We next evaluated its therapeutic potential in an epileptogenesis model. To better model epileptogenesis, we analyzed seizure activity via video-EEG monitoring for three consecutive days beginning at 28 days post-IHKA injection (Figure 2H). OICR-9429 treatment significantly reduced the frequency of SRS, although seizure duration remained unaffected (Figure 2I-K). Furthermore, to more comprehensively evaluate the therapeutic effects, we examined neuronal survival in the hippocampal CA1 region at 30 days post-IHKA injection, a hallmark pathology of this model. We found that OICR-9429 treatment significantly rescued the IHKA-induced neuronal loss in the CA1 area (Figure S3A, B). Collectively, these data revealed that pharmacological inhibition of the H3K4me3-catalyzing WDR5-KMT2 complex exerts antiepileptic effects.

### Neuron-specific knockdown of WDR5 in hippocampal neurons reduces the severity of epilepsy

**Figure 3:**
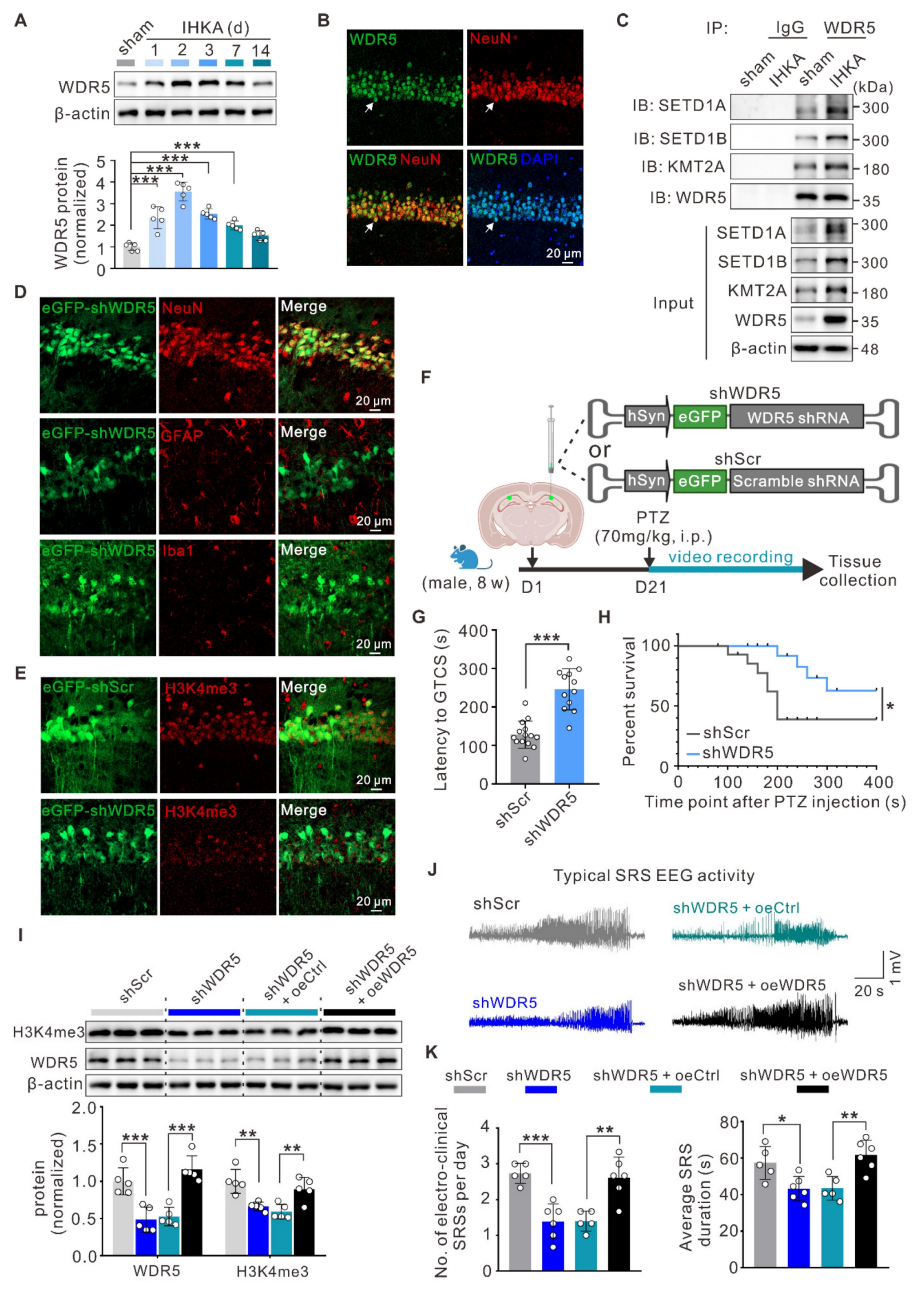
** Neuronal WDR5 knockdown diminishes H3K4me3 levels and suppresses epilepsy development. (A)** Representative immunoblots (top) and quantitative analysis (bottom) of WDR5 in the ipsilateral hippocampus following IHKA microinjection. n = 5 biologically independent mice per group. **(B)** Representative confocal images of the hippocampal CA1 from 2 d post-IHKA mice, stained for WDR5 (green), NeuN (red), and DAPI (blue).** (C)** Representative Co-IP analysis of WDR5 interactions with SETD1A, SETD1B, and KMT2A in hippocampal lysates from sham control and IHKA-2d mice. IP was performed with anti-WDR5 antibody; rabbit IgG served as negative control. IB: immunoblot. **(D)** Confocal microscopy validation of neuron-specific shWDR5 transduction efficiency in hippocampal CA1. Sections from shWDR5 transduced mice were co-stained with eGFP (green) and cell-type markers: NeuN (red), glial fibrillary acidic protein (GFAP; red), or induction of brown adipocytes 1 (Iba1; red). **(E)** Representative confocal images show hippocampal CA1 neurons transduced with either shScr or shWDR5 vectors, immunostained for eGFP (green) and H3K4me3 (red). **(F)** Experimental timeline. **(G)** Effects of conditional WDR5 knockdown on GTCS onset latency relative to shScr control. n = 15 biologically independent mice per group. One mouse in the shScr group and three in the shWDR5 group did not reach kindling criteria. **(H)** Kaplan-Meier survival curves of shScr and shWDR5 transduced mice following PTZ administration (70 mg/kg, i.p.). n = 15 biologically independent mice per group. **(I)** Representative immunoblots (top) and quantitative analysis (bottom) of H3K4me3 and WDR5 protein levels in hippocampal lysates from PTZ-kindled mice transduced with indicated AAVs. n = 5 biologically independent mice per group. **(J)** Representative EEG traces showing typical SRS electrographic activity recorded from chronic IHKA-TLE mice transduced with indicated AAVs. **(K)** Daily frequency (left) and mean duration (right) of electroclinical SRSs in chronic IHKA-TLE mice transduced with indicated AAVs. n = 6 biologically independent mice per group; one mouse in the shScr group died at 6 hours post-SE, and one mouse in the shWDR5+oeCtrl group died on the second day post-SE.*p < 0.05, **p < 0.01, ***p < 0.001. Statistical analyses were performed as follows: one-way ANOVA with Dunnett's post hoc test (Figure 3A); two-tailed unpaired Student's t-tests (Figure 3G); log-rank test (Figure 3H); one-way ANOVA with Tukey's post hoc test (Figure 3I, K).

To better understand the contribution of WDR5-mediated H3K4me3 modification to epileptogenesis, we systematically examined WDR5 expression patterns. WB analysis revealed time-dependent upregulation of WDR5 after IHKA injection, with an expression profile closely paralleling that of H3K4me3 (Figure 3A; Figure 1D). As expected, the expression level of WDR5 did not significantly change at different time points following sham surgery (Figure S2A). Furthermore, immunofluorescence staining demonstrated robust nuclear localization of WDR5 in the hippocampal neurons of IHKA-2d mice (Figure 3B), whereas only weak expression was observed in the nuclei of astrocytes and microglia (Figure S2B). The expression pattern of WDR5 during epileptogenesis, along with its prominent neuronal nuclear localization, closely mirrors that of H3K4me3. This correlation strongly suggests that WDR5-mediated H3K4me3 modification likely contributes to epileptogenesis in hippocampal neurons. To determine whether WDR5 exerts its function through the WDR5-KMT2 complex, we performed coimmunoprecipitation (Co-IP) assays. Compared with those in the sham group, the interactions between WDR5 and KMT2A, SETD1A, and SETD1B in IHKA-2d hippocampal tissues were significantly greater (Figure 3C). This increased assembly of the WDR5-KMT2 complex may be attributed to the elevated expression of both WDR5 and KMT2 family members in the hippocampus following epilepsy induction (Figure 3A; Figure 2B).

To evaluate whether neuron-specific WDR5 knockdown could normalize aberrant H3K4me3 and exert therapeutic effects in epileptic mice, we generated three adeno-associated viruses (AAVs) for neuronal knockdown of *Wdr5* (shWDR5 #1-3), each driven by the human synapsin 1 promoter (hSyn) for neuron-specific expression and tagged with an enhanced green fluorescent protein (eGFP) reporter. WB analysis at three weeks posttransduction confirmed the knockdown efficiency, and shWDR5 #3 (hereafter shWDR5), which demonstrated the most robust *in vivo* suppression, was selected for subsequent experiments (Figure S2C). Further immunofluorescence analysis confirmed the neuron-specific expression of shWDR5 in the hippocampal CA1 region (Figure 3D; Figure S2D). Moreover, in eGFP-positive neurons transduced with shWDR5, the fluorescence intensity of H3K4me3 was significantly lower than that in scramble control (shScr)-transduced neurons (Figure 3E, Figure S2E, F). Behavioral assessments using the PTZ acute kindling model at three weeks post-AAV administration revealed that WDR5 knockdown significantly prolonged the latency to GTCS and improved overall survival (Figure 3F-H). Nuclear protein analysis revealed that neuron-specific WDR5 knockdown, which mirrored pharmacological WDR5 inhibition, significantly decreased H3K4me3 levels without altering H3K27me3 levels (Figure S2G, H).

To rule out potential off-target effects of AAV-mediated WDR5 knockdown, we constructed a shRNA-resistant WDR5 rescue vector (oeWDR5) expressing full-length mouse Wdr5 under the hSyn promoter tagged with mCherry. A parallel hSyn-mCherry empty vector (oeCtrl) served as the control. Successful oeWDR5 transduction effectively restored both WDR5 expression and H3K4me3 levels, which were diminished by shWDR5 (Figure 3I; Figure S3C). Notably, in the IHKA-TLE model, oeWDR5 expression abrogated the shWDR5-mediated reduction in SRS frequency and duration (Figure 3J, K; Figure S3D). Collectively, these results demonstrate that neuron-targeted WDR5 knockdown reduces H3K4me3 modification and confers anti-epileptogenic effects.

### Neuron-targeted WDR5 knockdown ameliorates the E/I imbalance in the neural networks of epileptic mice

**Figure 4:**
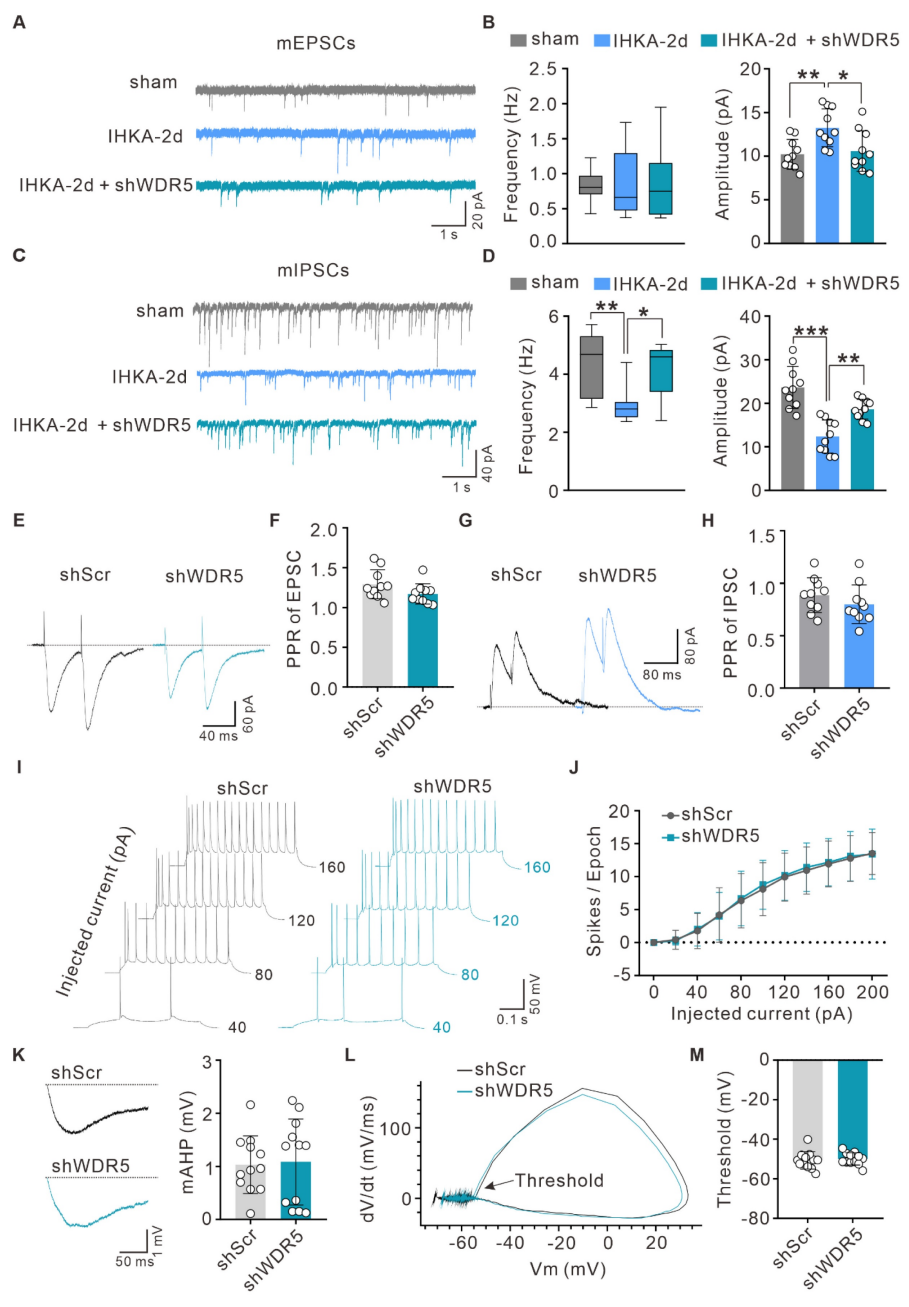
** WDR5 knockdown reduces neuronal hyperexcitability in epileptic hippocampus through synaptic transmission rather than intrinsic excitability. (A, C)** Representative traces of mEPSCs (**A**) and mIPSCs (**C**) recorded from hippocampal CA1 pyramidal neurons in acute brain slices of sham control mice and IHKA-induced epileptic mice (IHKA-2d) transduced with shScr or shWDR5. **(B, D)** Summary bar graphs showing the frequency and amplitude of mEPSCs (**B**) and mIPSCs (**D**). n = 10 cells from 3-4 mice per group. (**E, G**) PPR of evoked EPSCs (**E**) and IPSCs (**G**) recorded from hippocampal CA1 pyramidal neurons in IHKA-2d mice transduced with shScr or shWDR5. **(F, H)** Quantification of EPSC-PPR (**F**) and IPSC-PPR (**H**). n = 10 cells from 4 mice per group. *p < 0.05, **p < 0.01, ***p < 0.001. **(I)** Representative traces of AP firing patterns in CA1 pyramidal neurons from IHKA-2d mice transduced with shScr or shWDR5, evoked by 20 pA current steps. **(J)** Frequency-current (F-I) relationship quantified as spikes per 500-ms depolarizing epoch (as in a). n = 12 cells from 4 mice per group. **(K)** mAHP traces recorded in CA1 pyramidal neurons from IHKA-2d mice transduced with shScr or shWDR5. n = 12 cells from 4 mice per group. **(L)** Phase-plane plots of APs recorded from CA1 pyramidal neurons in IHKA-2d mice transduced with either shScr or shWDR5. n = 12 cells from 4 mice per group. **(M)** Quantification of AP threshold from the first spike evoked by near-rheobase current-injection in CA1 pyramidal neurons of IHKA-2d mice transduced with shScr or shWDR5. n = 12 cells from 4 mice per group. Statistical analyses were performed as follows: two-way repeated measures ANOVA (Figure 4J); two-tailed unpaired Student's t-tests (Figures 4F, H, K, M). In Figure 4B and 4D, frequency data were analyzed using the Kruskal-Wallis test with Dunn's post hoc test, while amplitude data were assessed with one-way ANOVA with Tukey's post hoc test.

Hyperexcitability of hippocampal CA1 pyramidal neurons is a hallmark of epilepsy 40, 41. To investigate the synaptic mechanisms underlying the antiepileptic effects of neuron-specific WDR5 knockdown, we first examined synaptic transmission in the hippocampal CA3-CA1 circuit. Compared to the sham group, hippocampal CA1 pyramidal neurons from IHKA-2d mice exhibited a significant increase in the amplitude, but not the frequency, of miniature excitatory postsynaptic currents (mEPSCs) (Figure 4A, B). Notably, WDR5 knockdown significantly reduced this aberrantly enhanced mEPSC amplitude without altering frequency in IHKA-2d mice (Figure 4A, B). Conversely, both the frequency and amplitude of miniature inhibitory postsynaptic currents (mIPSCs) were significantly reduced in IHKA-2d mice compared to the sham controls (Figure 4C, D). Importantly, WDR5 knockdown markedly increased both mIPSC frequency and amplitude in the epileptic condition (Figure 4C, D). These results indicated that WDR5 knockdown modulates both excitatory and inhibitory synaptic transmission.

To determine whether WDR5 acts by altering the probability of presynaptic neurotransmitter release, we assessed the paired-pulse ratio (PPR) in IHKA-2d mice with or without WDR5 knockdown. We found that the PPR of both evoked EPSCs and IPSCs remained unchanged across groups (Figure 4E-H), suggesting that presynaptic release probability was not significantly affected and that WDR5 likely exerts its effects primarily at postsynaptic sites. We next assessed potential alterations in the intrinsic properties of CA1 pyramidal neurons in IHKA-2d mice affected by WDR5 knockdown. Whole-cell current-clamp recordings revealed no significant differences in intrinsic excitability between WDR5-knockdown and control IHKA-2d mice, as quantified by current-firing curves and rheobases during the depolarizing current steps (Figure 4I, J; Figure S4A). Furthermore, the medium afterhyperpolarization (mAHP), action potential (AP) threshold, input resistance, resting membrane potential (RMP), AP peak, and membrane capacitance were not altered in WDR5-knockdown IHKA-2d CA1 pyramidal neurons (Figure 4K-M; Figure S4B-F), indicating that intrinsic neuronal excitability remains unchanged. Collectively, these results demonstrate that neuron-targeted WDR5 knockdown preferentially modulates postsynaptic mechanisms to rebalance E/I synaptic transmission and alleviate network hyperexcitability in epilepsy.

### Increased WDR5-H3K4me3 levels are responsible for the increase in ARID5B transcription under epileptic conditions

**Figure 5:**
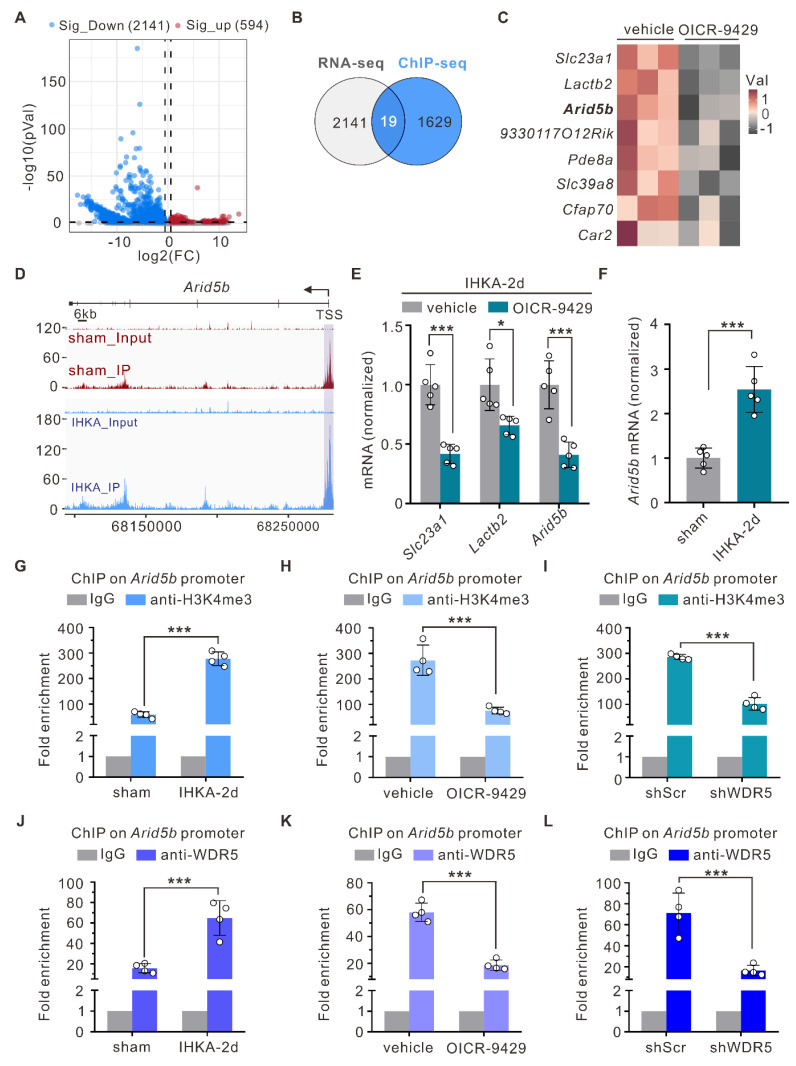
***Arid5b* as a potential direct target of WDR5-H3K4me3 under epileptic condition*.* (A)** Volcano plot of RNA-seq analysis showing DEGs in the ipsilateral hippocampus of IHKA-2d mice pretreated with vehicle control or OICR-9429. Mice received intraperitoneal injections of OICR-9429 or vehicle control every 24 hours beginning 3 days prior to IHKA induction. Hippocampal tissues were collected for RNA-seq 2 days post-modeling. n = 3 biologically independent mice per group. Cutoff criteria: fold change > 1.5, P < 0.05. FC, fold change.** (B)** Venn diagram showing overlapping genes between H3K4me3-enriched genes in IHKA-2d mice and genes with reduced expression following OICR-9429 treatment. **(C)** Heatmap displaying the top 8 most significantly downregulated genes selected from the 19 overlapping genes identified in (**B**). **(D)** Genome browser snapshots of H3K4me3 ChIP-seq signals at the *Arid5b* locus in sham control versus IHKA-2d groups. Purple shading highlights H3K4me3 peak enrichment at the *Arid5b* promoter region. **(E)** qPCR analysis of indicated mRNA expression levels in ipsilateral hippocampal tissues IHKA-2d mice pretreated with vehicle control or OICR-9429. n=5 biologically independent mice per group.** (F)** qPCR analysis of *Arid5b* mRNA expression in ipsilateral hippocampal tissues from sham control versus IHKA-2d mice. n=5 biologically independent mice per group. **(G-I)** ChIP-qPCR analysis of H3K4me3 enrichment at the *Arid5b* promoter in hippocampal tissues from indicated groups. Data represent fold-enrichment over IgG control calculated by 2^-ΔΔCT^ method. n = 4 biologically independent mice per group. **(J-L)** ChIP-qPCR analysis of WDR5 enrichment at the *Arid5b* promoter in hippocampal tissues from indicated groups. n = 4 biologically independent mice per group. *p < 0.05, ***p < 0.001. Statistical analyses were performed as follows: two-tailed unpaired Student's t-tests with Holm-Šidák correction for multiple comparisons (Figure 5E); two-tailed unpaired Student's t-tests (Figure 5F); one-way ANOVA with Dunnett's post hoc test (Figure 5G-L).

To elucidate how elevated WDR5-H3K4me3 levels drive epileptogenesis, we next performed RNA sequencing (RNA-seq) on hippocampal tissues from IHKA-2d mice with or without OICR-9429 treatment and systematically analyzed the impact of WDR5-KMT2 complex inhibition on the hippocampal transcriptome. Compared with vehicle control mice, OICR-9429-treated IHKA-2d mice presented 2,735 differentially expressed genes (DEGs), with 594 significantly upregulated genes and 2,141 significantly downregulated genes (Figure 5A). Since H3K4me3 is considered a mark of active transcription, genes whose expression decreases following WDR5-KMT2 complex inhibition are more likely to represent direct targets. Integration of RNA-seq and ChIP-seq (sham vs. IHKA-2d) data revealed 19 genes that exhibited both increased H3K4me3 levels in IHKA-2d mice and decreased transcript levels after WDR5-KMT2 complex inhibition (Figure 5B). Bioinformatic analysis revealed solute carrier family 23 (nucleobase transporters), member 1 (*Slc23a1*), lactamase, beta 2 (*Lactb2*), and *Arid5b* as the top three genes showing both reduced expression after WDR5 inhibition (Figure 5C) and increased H3K4me3 occupancy at promoter regions under epileptic conditions (Figure 5D; Figure S5). qPCR validation confirmed significantly reduced transcript levels of these genes in OICR-9429-treated IHKA-2d hippocampal tissues, which was consistent with the RNA-seq results (Figure 5E). Among these genes,* Arid5b* encodes the ARID5B protein, which was initially cloned as a transcriptional regulator that binds to gene promoter regions 42, 43. ARID5B has been demonstrated to exhibit dual functions in both transcriptional repression and activation 44, 45. This finding aligns with the ChIP-seq data showing that genes with altered H3K4me3 modification during epileptogenesis were most significantly enriched in transcriptional regulation biological processes (Figure 1I). Interestingly, a previous study in patients suggested a potential association of *Arid5b* with epilepsy 46, suggesting that *Arid5b* may be a direct target of WDR5-H3K4me3 under epileptic conditions.

To further demonstrate that *Arid5b* is regulated by the WDR5-KMT2 complex under epileptogenic conditions, we first performed qPCR analysis and found that *Arid5b* expression was significantly upregulated in the hippocampal tissues of IHKA-2d mice (Figure 5F). This increased expression correlated with elevated levels of both WDR5 and H3K4me3 (Figure 1E; Figure 3A). The ChIP‒qPCR results confirmed the ChIP‒seq data, which revealed significantly increased H3K4me3 occupancy at the *Arid5b* promoter region in the IHKA-2d mouse hippocampus (Figure 5G). Furthermore, ChIP‒qPCR analysis revealed reduced H3K4me3 enrichment at the *Arid5b* promoter following OICR-9429 treatment compared with the control (Figure 5H). AAV-mediated neuron-specific knockdown of WDR5 produced effects consistent with those of OICR-9429 treatment (Figure 5I). Additionally, we detected increased WDR5 occupancy at the *Arid5b* promoter in the IHKA-2d mouse hippocampus (Figure 5J), whereas pharmacological inhibition or neuron-specific WDR5 knockdown reduced WDR5 enrichment at this locus (Figure 5K, L). These results demonstrate that elevated WDR5-H3K4me3 levels drive increased *Arid5b* transcription during epileptogenesis.

### Neuron-specific knockdown of ARID5B alleviates neuronal hyperexcitability and attenuates seizure severity

**Figure 6:**
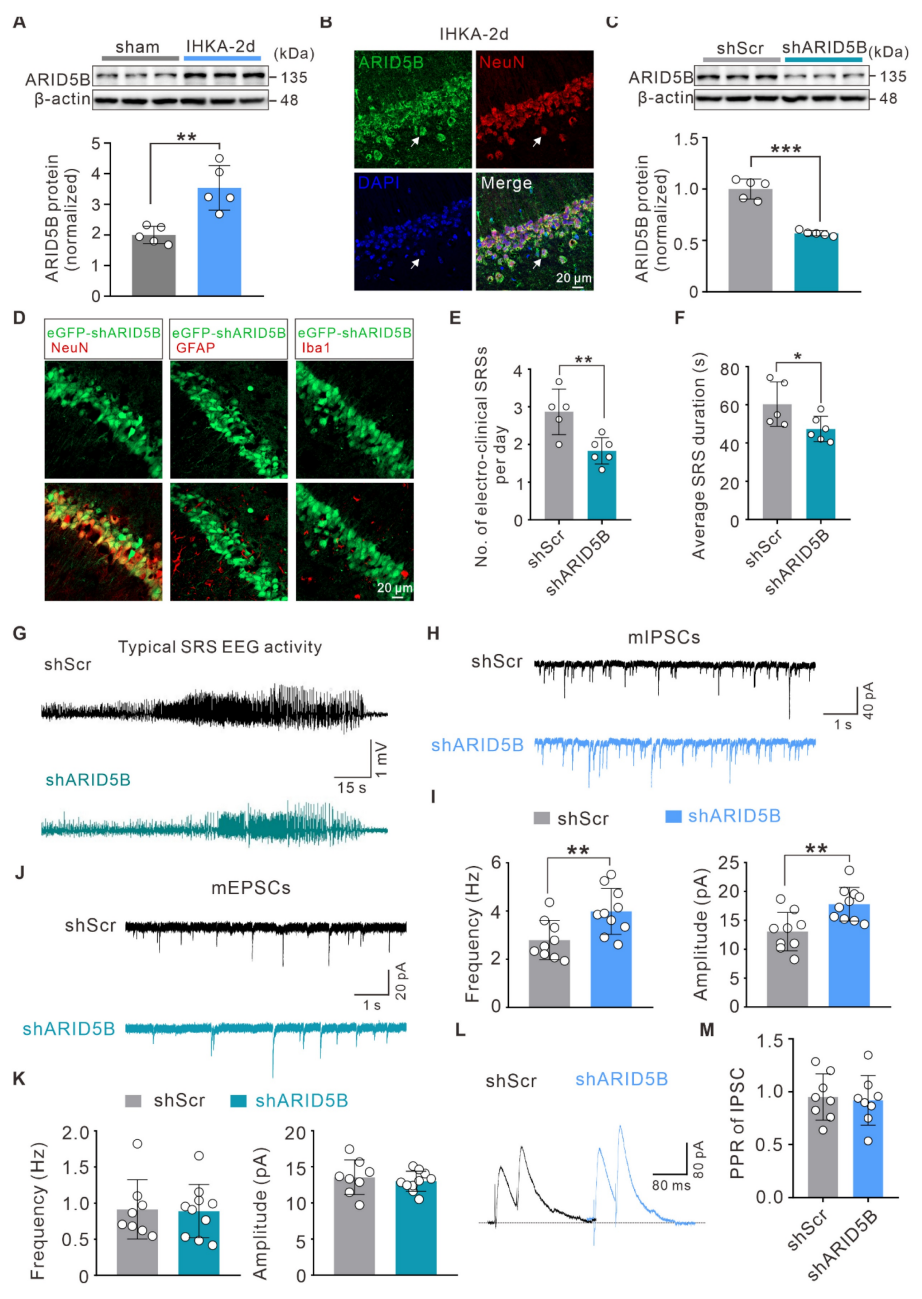
** Neuron-specific knockdown of upregulated ARID5B in IHKA-2d hippocampus enhances inhibitory synaptic transmission and alleviates seizure severity. (A)** Representative immunoblots (top) and quantitative analysis (bottom) of ARID5B protein expression in ipsilateral hippocampal tissues from sham control versus IHKA-2d mice. n = 5 biologically independent mice per group. **(B)** Representative confocal images of the hippocampal CA1 from 2 d post-IHKA mice, stained for ARID5B (green), NeuN (red), and DAPI (blue). **(C)** Representative immunoblots (top) and quantitative analysis (bottom) of ARID5B protein in hippocampal lysates from mice transduced with shScr or shARID5B. n = 5 biologically independent mice per group. **(D)** Confocal microscopy validation of neuron-specific shARID5B-AAV transduction efficiency in hippocampal CA1. Sections from shARID5B-AAV-eGFP transduced mice were co-stained with eGFP (green) and cell-type markers: NeuN (red), GFAP (red), or Iba1 (red). **(E-F)** Bar graph denotes electroclinical SRS (**E**) daily frequency and (**F**) mean duration in chronic IHKA-TLE mice transduced with shScr or shARID5B. n = 7 biologically independent mice per group. One mouse in the shARID5B group died during the acute phase and was excluded. In the shScr group, one mouse died on day 6 post-SE, and one mouse that developed SE did not exhibit SRSs during the chronic monitoring period and was therefore excluded from analysis. **(G)** Representative EEG traces showing typical SRS electrographic activity recorded from chronic IHKA-TLE mice transduced with either shScr or shARID5B.** (H, J)** Representative traces of mIPSCs (**H**) and mEPSCs (**J**) recorded from hippocampal CA1 pyramidal neurons in acute brain slices of IHKA-2d mice transduced with shScr or shARID5B. **(I, K)** Summary bar graphs showing the frequency and amplitude of mIPSCs (I) and mEPSCs (K). n= 8-10 cells from 3-4 mice per group. **(L)** PPR of evoked IPSCs recorded from hippocampal CA1 pyramidal neurons in IHKA-2d mice transduced with shScr or shARID5B. **(M)** Quantification of IPSC-PPR. n= 8 cells from 3-4 mice per group. *p < 0.05, **p < 0.01, ***p < 0.001. Statistical comparisons were performed using two-tailed unpaired Student's t-tests.

To elucidate the role of ARID5B in epilepsy, we first investigated whether ARID5B protein expression is altered. WB analysis revealed a significant increase in ARID5B protein levels in the hippocampi of IHKA-2d mice compared with those in sham control mice (Figure 6A). Furthermore, ARID5B exhibited prominent localization in hippocampal neurons (Figure 6B), which was consistent with the expression patterns of WDR5 and H3K4me3 (Figure 1F; Figure 3B). To investigate the effect of neuronal ARID5B modulation on epileptic phenotypes, we generated AAV vectors carrying hSyn promoter-driven neuron-specific ARID5B-knockdown constructs (shARID5B). The knockdown efficiency was confirmed by WB (Figure 6C) and immunofluorescence (Figure 6D). Behavioral analysis of IHKA-TLE revealed that neuron-specific ARID5B knockdown significantly reduced the SRS frequency and duration compared with those in the control group (Figure 6E-G). To assess whether ARID5B knockdown induces synaptic transmission changes consistent with those resulting from WDR5 knockdown, we performed whole-cell patch-clamp recordings. Neuron-specific ARID5B knockdown resulted in a significant increase in both the frequency and amplitude of mIPSCs in the hippocampal CA1 pyramidal neurons of IHKA-2d mice (Figure 6H, I), which aligns with the effects of WDR5 knockdown on inhibitory synapses (Figure 4C, D). Surprisingly, ARID5B knockdown did not alter the amplitude or frequency of mEPSCs (Figure 6J, K). Additionally, ARID5B knockdown had no significant effect on IPSC-PPR (Figure 6L, M), suggesting that neuron-specific ARID5B knockdown may primarily shift the E/I balance toward reduced excitability through postsynaptic inhibitory mechanisms, thereby attenuating the epileptic phenotype.

### The neuronal WDR5-ARID5B axis enhances seizure susceptibility by decreasing GABA_A_R α1 and α2 subunit expression

**Figure 7:**
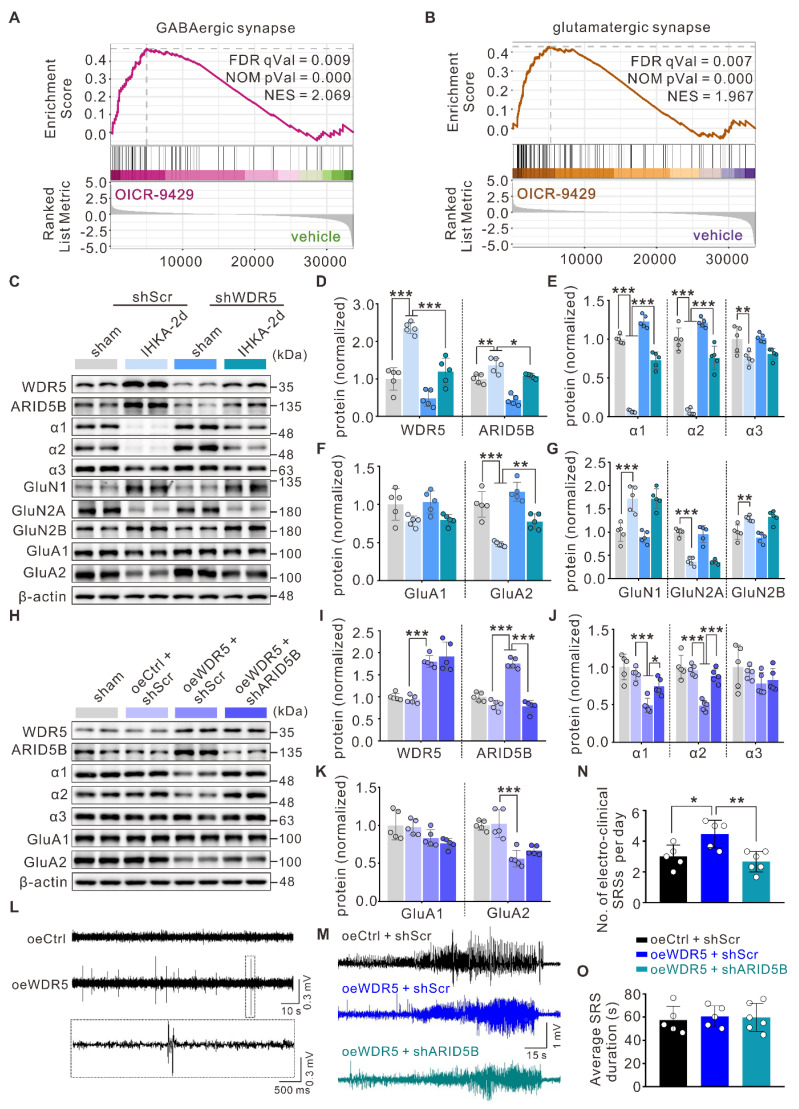
** ARID5B is essential for WDR5-mediated regulation of epileptogenesis through inhibitory synaptic receptors. (A-B)** GSEA enrichment plot of the GABAergic (**A**) and glutamatergic (**B**) synapse pathway showing significant differential enrichment in vehicle- versus OICR-9429-pre-treated IHKA-2d mice. **(C-G)** Representative immunoblots (**C**) and quantitative analysis (**D-G**) of WDR5, ARID5B and other synaptic protein levels in ipsilateral hippocampal lysates from indicated groups. n = 5 biologically independent mice per group.** (H-K)** Representative immunoblots (**H**) and quantitative analysis (**I-K**) of WDR5, ARID5B and other synaptic protein levels in hippocampal lysates from indicated groups. n = 5 biologically independent mice per group.** (L)** Representative EEG traces: (top) Control (oeCtrl) mouse showing physiological activity; (bottom) oeWDR5 mouse exhibiting spike waves. The boxed region indicates the magnified epileptiform discharges. **(M)** Representative EEG traces showing typical SRS electrographic activity recorded from chronic IHKA-TLE mice transduced with indicated AAVs.** (N-O)** Daily frequency (N) and mean duration (O) of electroclinical SRSs in chronic IHKA-TLE mice transduced with indicated AAVs. n = 7 biologically independent mice per group, following the exclusion of mortalities in oeCtrl+shScr (2 mice: 1d and 20d post-SE), oeWDR5+shScr (2 mice: 1d and 4d post-SE), and oeWDR5+shARID5B (1 mouse: 2d post-SE). *p < 0.05, **p < 0.01, ***p < 0.001. Statistical comparisons were performed using one-way ANOVA with Tukey's post hoc test.

Our whole-cell patch-clamp data demonstrated that both WDR5 and ARID5B affect epileptic phenotypes primarily through postsynaptic mechanisms. This conclusion was further corroborated by gene set enrichment analysis (GSEA) of our RNA-seq data, which revealed strong enrichment of pathways related to GABAergic and glutamatergic synaptic transmission in the DEGs (Figure 7A, B). Altered expression and function of inhibitory GABA_A_R subunits (α1-3), excitatory N-methyl-D-aspartate receptor (NMDAR) subunits (GluN1, GluN2A, GluN2B), and AMPAR subunits (GluA1, GluA2) have been demonstrated to be associated with E/I imbalance in epilepsy 47, 48. We therefore hypothesized that the WDR5-ARID5B axis might ultimately affect the expression of certain receptor subunits through direct or indirect mechanisms, thereby influencing epileptic phenotypes. To test this hypothesis, we first examined the effect of blocking WDR5 upregulation in IHKA-2d hippocampal neurons on ARID5B and postsynaptic receptor protein expression. We found that neuronal-specific knockdown of WDR5 abolished the IHKA-2d-induced increase in WDR5 and ARID5B protein levels (Figure 7C, D) and partially abrogated the downregulation of the α1, α2, and GluA2 subunits (Figure 7C-F) but had no significant effect on the α3, GluA1, or NMDAR subunits (Figure 7C-E, G). Similar effects were observed in KA-treated cultured primary hippocampal neurons following WDR5 knockdown (Figure S6 and Figure S7). As previous studies have reported that ARID5B functions as a bidirectional transcriptional regulator, combined with our findings, we propose that WDR5 may negatively regulate α1, α2, and GluA2 subunit expression by stabilizing ARID5B.

To rule out the possibility that WDR5 regulates α1, α2, and GluA2 through direct or alternative pathways, we overexpressed full-length WDR5 via AAV transduction in the right hippocampus of naïve adult mice to mimic the WDR5 upregulation observed in the epileptic mouse hippocampus. WB analysis confirmed that WDR5 overexpression increased ARID5B levels while decreasing α1, α2, and GluA2 subunit expression (Figure 7H-K).

However, concurrent knockdown of ARID5B abrogated the WDR5-induced reduction in α1 and α2 levels but not GluA2 levels (Figure 7H-K). These findings indicate that ARID5B serves as an essential protein through which WDR5 specifically modulates inhibitory, but not excitatory, synaptic transmission. We next asked whether WDR5 overexpression alone could induce epileptic phenotypes. Video-EEG recordings in non-epileptic mice showed that it significantly increased subthreshold epileptiform activity (Figure 7L). This suggests a heightened state of network excitability that was insufficient, on its own, to induce seizures. To further elucidate the impact of the WDR5-ARID5B axis on epileptic phenotypes, we employed AAV-mediated gene manipulation in the IHKA model for behavioral analysis. Our results demonstrated that WDR5 overexpression significantly increased the frequency of SRS in IHKA-TLE model mice, although it did not significantly affect SRS duration (Figure 7M-O). Furthermore, ARID5B knockdown effectively abrogated the WDR5 overexpression-induced increase in SRS frequency (Figure 7M-O). Postbehavioral WB analysis of hippocampal tissues revealed that concurrent WDR5 overexpression and ARID5B knockdown rescued the reduced protein levels of the α1 and α2 subunits caused by WDR5 overexpression (Figure S8). These findings demonstrate that ARID5B is required for the WDR5-mediated regulation of inhibitory synaptic receptor proteins in epileptogenesis.

### The neuronal WDR5-ARID5B axis contributes to epileptogenesis by impairing GABA_A_R subunit surface expression and inhibitory synaptic transmission

**Figure 8:**
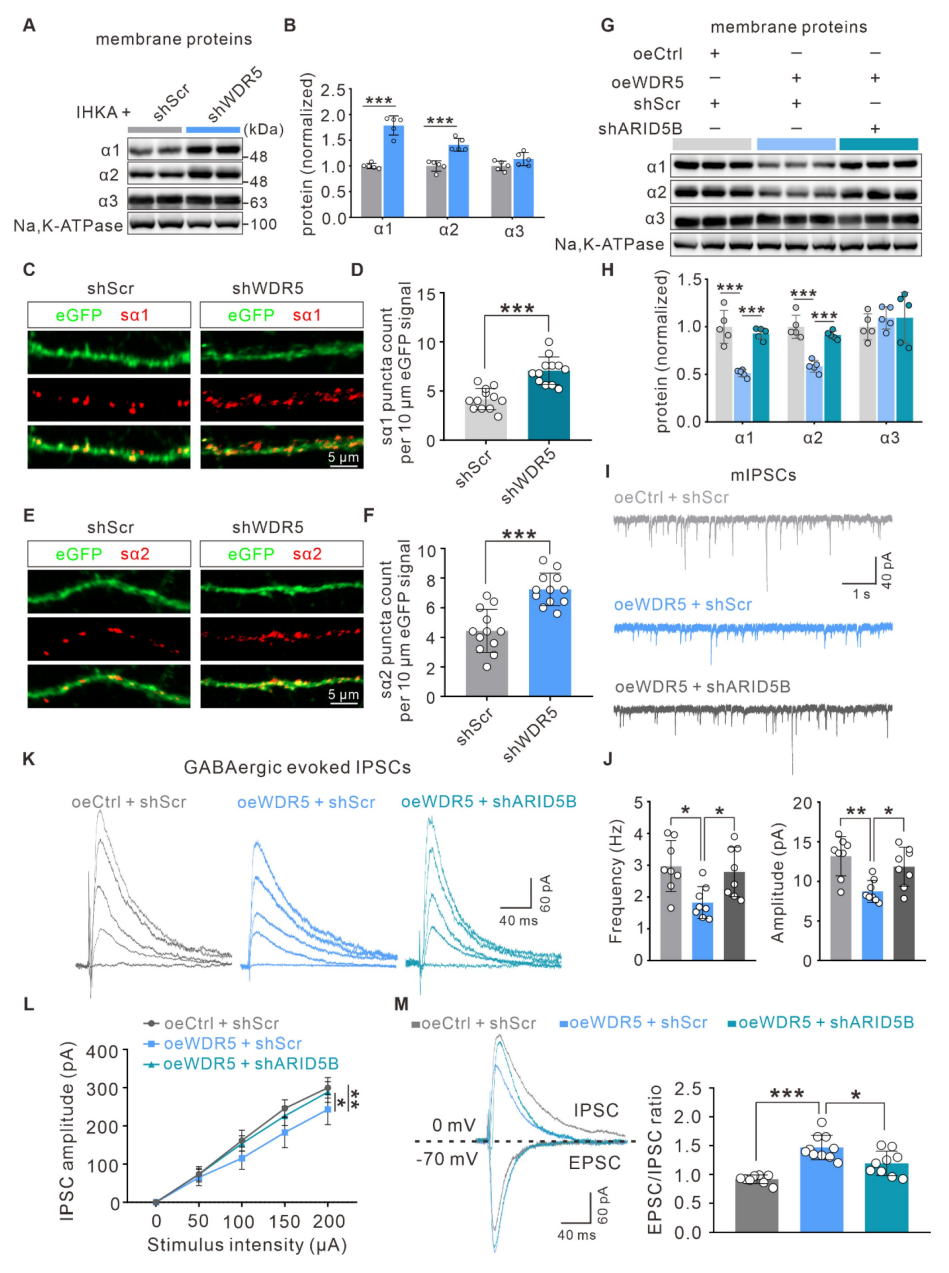
** ARID5B knockdown restores GABAergic synaptic transmission impaired by WDR5 overexpression. (A-B)** Representative immunoblots (**A**) and quantitative analysis (**B**) of surface α1, α2, and α3 subunit protein levels in hippocampal membrane lysates from IHKA-2d mice transduced with shScr or shWDR5. Sodium Potassium ATPase (Na,K-ATPase) was used as a plasma loading control. n = 5 biologically independent mice per group. **(C-F)** Representative confocal images (**C, E**) and quantitative analysis (**D, F**) of synaptic surface GABA_A_R α1 subunit (sα1) and α2 subunit (sα2) clusters in cultured hippocampal neurons infected at DIV3 with shCtrl or shWDR5. Neurons were treated with KA (10 μM) for 24 h at DIV14, followed by immunostaining with antibodies against sα1 or sα2 (red). n = 12 neurons from 3 independent cultures per group. **(G, H)** Representative immunoblots (**G**) and quantitative analysis (**H**) of surface α1, α2, and α3 subunit protein levels in hippocampal membrane lysates from mice transduced with shScr or shWDR5. n = 5 biologically independent mice per group. **(I)** Representative traces of mIPSCs recorded from hippocampal CA1 pyramidal neurons in acute brain slices of mice transduced with indicated AAVs.** (J)** Summary bar graphs showing the frequency and amplitude of mIPSCs. n = 8 cells from 4 mice per group. **(K)** Evoked IPSCs recorded from hippocampal CA1 pyramidal neurons in response to incremental stimulus intensities in mice transduced with indicated AAVs. **(L)** Input-output relationships quantified as eIPSC amplitude versus stimulus intensity. n = 7-8 cells from 3 mice per group. **(M)** Left: Representative traces of the evoked IPSCs (top) and EPSCs (bottom) recorded from the same hippocampal CA1 pyramidal neurons of mice transduced with indicated AAVs. Right: Quantification of the EPSC/IPSC ratio (peak amplitude ratio). n = 8-10 cells from 4 mice per group. *p < 0.05, **p < 0.01, ***p < 0.001. Statistical analyses were performed as follows: two-tailed unpaired Student's t-tests with Holm-Šidák correction for multiple comparisons (Figure 8B); two-tailed unpaired Student's t-tests (Figure 8D, F); one-way ANOVA with Tukey's post hoc test (Figure 8H, J); two-way repeated measure ANOVA (Figure 8L); Welch's ANOVA with Games-Howell post hoc tests (Figure 8M).

To determine whether WDR5-ARID5B-mediated alterations in total GABA_A_R α1 and α2 subunit proteins further influence membrane protein levels and functional outcomes, we first examined their surface expression. Consistent with total protein levels, surface expression of the α1 and α2 subunits was increased in the hippocampi of IHKA-2d model mice with WDR5 knockdown (Figure 8A, B). Using a cell-impermeant live immunostaining assay with antibodies targeting the extracellular N-terminal domains of α1 and α2, we further assessed their surface expression on dendrites of cultured primary neurons. Notably, surface clusters of α1 (sα1) and α2 (sα2) were significantly elevated in WDR5-knockdown, KA-treated day *in vitro* 15 (DIV15) primary hippocampal neurons (Figure 8C-F).

To investigate whether ARID5B could reverse WDR5 overexpression-induced synaptic changes, we analyzed membrane protein levels and found that neuron-specific ARID5B knockdown abrogated the reduced surface expression of the α1 and α2 subunits caused by WDR5 overexpression (Figure 8G, H). To determine whether WDR5-ARID5B-GABA_A_R axis alterations translate into functional synaptic changes, we performed whole-cell patch-clamp recordings to assess neurotransmission at excitatory CA3‒CA1 synapses. ARID5B knockdown significantly abrogated the decreased frequency and amplitude of mIPSCs induced by WDR5 overexpression (Figure 8I, J). Furthermore, evoked IPSC recordings revealed that WDR5 overexpression substantially reduced IPSC amplitudes across a range of stimulus intensities, whereas ARID5B knockdown restored these amplitudes (Figure 8K, L). Additionally, WDR5 overexpression led to a significant increase in the EPSC/IPSC ratio, which was abrogated by ARID5B knockdown (Figure 8M). These findings suggest that in epilepsy, elevated WDR5 expression impairs GABAergic synaptic transmission in hippocampal neurons, likely by upregulating ARID5B expression, which subsequently downregulates GABA_A_R subunits at both the total and membrane protein levels.

## Discussion

TLE, the most prevalent form of drug-resistant epilepsy, is characterized by complex molecular processes such as ion channel dysfunction and epigenetic dysregulation 8, 49. The progression of epileptogenesis coincides with dynamic changes in brain gene expression profiles, where epigenetic mechanisms play crucial regulatory roles 6, 8. Previous epigenetic research in epilepsy has focused primarily on DNA methylation and histone acetylation 50-52. Notably, histone acetylation and deacetylation regulate the expression of numerous epilepsy-associated genes, and histone deacetylase inhibition has emerged as a promising epigenetic therapeutic strategy, with several inhibitors demonstrating efficacy in preventing epileptogenesis in animal models 52, 53. In contrast, the role of specific methylation marks such as H3K4me3 remains less explored. Our study therefore provides the first systematic analysis of genome-wide H3K4me3 modifications during epileptogenesis, demonstrating that H3K4me3 functions as a critical transcriptional regulator and revealing a neuron-specific WDR5-ARID5B-GABA_A_R epigenetic axis that drives TLE pathogenesis.

Global H3K4me3 dysregulation has been extensively documented in oncogenesis and neurodegenerative pathologies, including Alzheimer's disease 54, 55, yet its potential role in epileptogenesis remains unexplored. Zhang et al. reported upregulated *Kcnj10* expression in the hippocampus of pilocarpine-induced epileptic rats, potentially regulated by H3K9me2 modification 20. In contrast, our study focused on global alterations in histone methylation, revealing a significant increase in overall H3K4me3 levels during epileptogenesis. Elevated H3K4me3 levels induced broad genomic distribution characteristics, suggesting its capacity to orchestrate a more complex transcriptional network during epilepsy development. Furthermore, H3K4me3 levels returned to near baseline levels by 14 days post-IHKA injection (Figure 1D), which may reflect massive neuronal loss at this stage or, alternatively, indicate the primary involvement of H3K4me3 in epileptogenesis rather than seizure maintenance. Beyond its role as an acute response marker, the persistent H3K4me3 remodeling we observed raises the intriguing possibility that such acquired epigenetic alterations could establish a sustained hyperexcitable state, thereby predisposing neural circuits to seizure susceptibility. While our study does not directly address inherited epigenetic marks, the sustained dysregulation of the WDR5/H3K4me3 axis described herein may represent a form of "epigenetic memory" that contributes to long-term epilepsy vulnerability. Intriguingly, unlike H3K4me3, the repressive marker H3K27me3 displayed the most pronounced reduction in expression at IHKA-14d (Figure 1C), suggesting a possible role in seizure maintenance—a hypothesis that warrants further investigation. Furthermore, while our study delineates the role of H3K4me3, the dynamics of repressive marks such as H3K27me3 at earlier time points and their potential interaction with activating marks remain to be fully elucidated, which could further refine the epigenetic therapeutic window in epilepsy.

Notably, consistent with elevated H3K4me3 levels, we observed significant upregulation of KMT2 family proteins (KMT2A, SETD1A, and SETD1B) at both the transcript and protein levels in the hippocampi of epileptic mice (Figure 2A, B). These findings suggest that increased H3K4me3 levels in epilepsy likely results from coordinated regulation by multiple KMT2 members. To circumvent potential compensatory effects from targeting individual KMT2 proteins, we focused on the scaffold proteins that determine the activity of KMT2 methyltransferase complexes. It is well established that functionally intact KMT2 methyltransferases require the core scaffold protein WDR5, which recruits and stabilizes other complex components (ASH2L, RBBP5, and DPY30) through interactions with KMT2 proteins 56, 57. To our knowledge, the roles of these four scaffold proteins in TLE remain unreported. Intriguingly, a recent study identified epilepsy as a highly penetrant phenotype in 11 unrelated individuals carrying WDR5 missense mutations, suggesting potential dominant-negative or gain-of-function mechanisms 58. These findings prompted us to focus on the role of WDR5 in epileptogenesis. Strikingly, WDR5 expression followed a temporal pattern congruent with H3K4me3 upregulation post-IHKA injection (Figure 3A), while Co-IP assays revealed increased binding between WDR5 and KMT2A/SETD1A/SETD1B in epileptic tissues (Figure 3C). These findings indicate that increased WDR5-KMT2 complex activity during early epileptogenesis is likely attributable to increased expression of its constituent proteins. Notably, our findings suggest that neurons are likely the primary cellular targets mediating WDR5-H3K4me3-dependent epileptogenesis. While detectable WDR5/H3K4me3 expression was detected in astrocytes and microglia, their expression levels were substantially lower than those in neurons. This cell type-specific distribution implies distinct epigenetic regulatory mechanisms across neural populations. However, we cannot exclude potential contributions of the WDR5-ARID5B axis to epileptogenesis through its ability to modulate glial cell function.

Integrated analysis of ChIP-seq and RNA-seq data coupled with functional validation identified ARID5B as a critical mediator of WDR5-H3K4me3-dependent pro-epileptogenic effects in hippocampal neurons (Figure 5). ARID5B, a transcriptional regulator containing the ARID/Bright domain, has been reported to function as both a sequence-specific and nonspecific DNA-binding domain 43. Previous studies have established the dual role of ARID5B in transcriptional repression and activation 44, 45. Our work provides the first evidence that ARID5B is essential for epileptogenesis. Importantly, we discovered that ARID5B selectively regulates the expression of GABA_A_R subunits but not that of glutamate receptor subunits during epileptogenesis. The observed subunit-specific regulation of GABA_A_Rs by ARID5B could be mediated by either direct binding to distinct DNA sequence motifs or indirect mechanisms, such as the transcriptional control of other associated genes (e.g., auxiliary GABA_A_R subunits) or the recruitment of coregulators. The precise molecular mechanism remains to be elucidated. Overall, our study reveals a novel mechanism whereby H3K4me3-mediated epigenetic information is translated into altered inhibitory synaptic protein expression via ARID5B, thereby expanding our understanding of how the "histone code" orchestrates neural circuit regulation in epilepsy pathogenesis.

Neuronal circuit function requires precise coordination between synaptic excitation and inhibition, and disruption of the E/I balance promotes epileptogenesis 59, 60. Our study demonstrated that the neuronal WDR5-ARID5B axis drives epileptogenesis by impairing GABA_A_R-mediated inhibitory synaptic transmission. As the primary inhibitory receptors in the CNS and key targets of antiepileptic drugs, GABA_A_Rs play pivotal roles in both the initiation and maintenance of epilepsy 61, 62. Intriguingly, we found that WDR5 additionally modulates glutamatergic synaptic function, yet ARID5B knockdown failed to rescue these excitatory synaptic effects, suggesting that WDR5 may regulate excitatory transmission through ARID5B-independent mechanisms. AMPARs, heterotetrameric ligand-gated channels composed of GluA1-4 subunits, mediate fast excitatory transmission 63. Notably, GluA2-lacking AMPARs exhibit Ca^2+^ permeability and contribute to neuronal hyperexcitability in epilepsy 63, 64. Our data show that WDR5 knockdown reduces mEPSC/eEPSC amplitudes. Combined with its negative regulation of GluA2 expression, we propose that WDR5 knockdown in epileptic hippocampal neurons may indirectly promote synaptic GluA2 incorporation, thereby replacing Ca^2+^-permeable GluA2 lacking AMPARs and decreasing receptor conductance, ultimately manifesting as reduced mEPSC/eEPSC amplitudes.

In contrast to conventional antiepileptic drugs that typically target single ion channels 65, drugs that target the WDR5-H3K4me3 axis may concurrently mitigate the dysregulation of multiple epilepsy-associated genes, thereby offering a novel therapeutic strategy to overcome the limitations of single-target approaches. Several limitations in this study warrant further investigation: (1) the current findings are primarily confined to hippocampal neurons within the CA1 subregion; a systematic evaluation of other hippocampal subfields (e.g., DG, CA3), additional epileptogenic regions (e.g., cortex), and non-neuronal cell types (e.g., astrocytes) is necessary for a comprehensive understanding; (2) the potential contributions of non-neuronal cells, given the observed low but detectable levels of WDR5 and H3K4me3 in glial nuclei (Supplementary Figs. 1B-C, 2B), the functional significance of which remains unclear; (3) the precise molecular mechanisms underlying WDR5-mediated excitatory synaptic regulation and ARID5B subunit-specific control of GABA_A_Rs remain to be elucidated; (4) the conservation and therapeutic relevance of this pathway in human TLE merit further validation; and (5) the exclusive use of male mice limits the generalizability of our findings to females. Furthermore, (6) it should be noted that our findings primarily inform epileptogenesis following severe SE, as the experimental design specifically selected animals meeting robust SE criteria. While this approach ensures a well-defined and homogeneous injury level for mechanistic investigation, future studies examining models with varying insult severity will be valuable to determine whether this epigenetic axis plays a universal or context-dependent role in epilepsy development. Given the well-established, bidirectional interplay between sex hormone fluctuations and epilepsy, which influences seizure mechanisms and antiseizure drug efficacy while posing unique clinical challenges 66-70, future studies should systematically examine these epigenetic mechanisms across different estrous stages to facilitate the development of sex-informed therapeutic strategies. Future studies utilizing cell-type-specific approaches in glial cells will be essential to dissect their unique contributions and to determine whether combinatorial targeting of both neuronal and glial epigenetic mechanisms could yield superior therapeutic outcomes.

**Figure 9:**
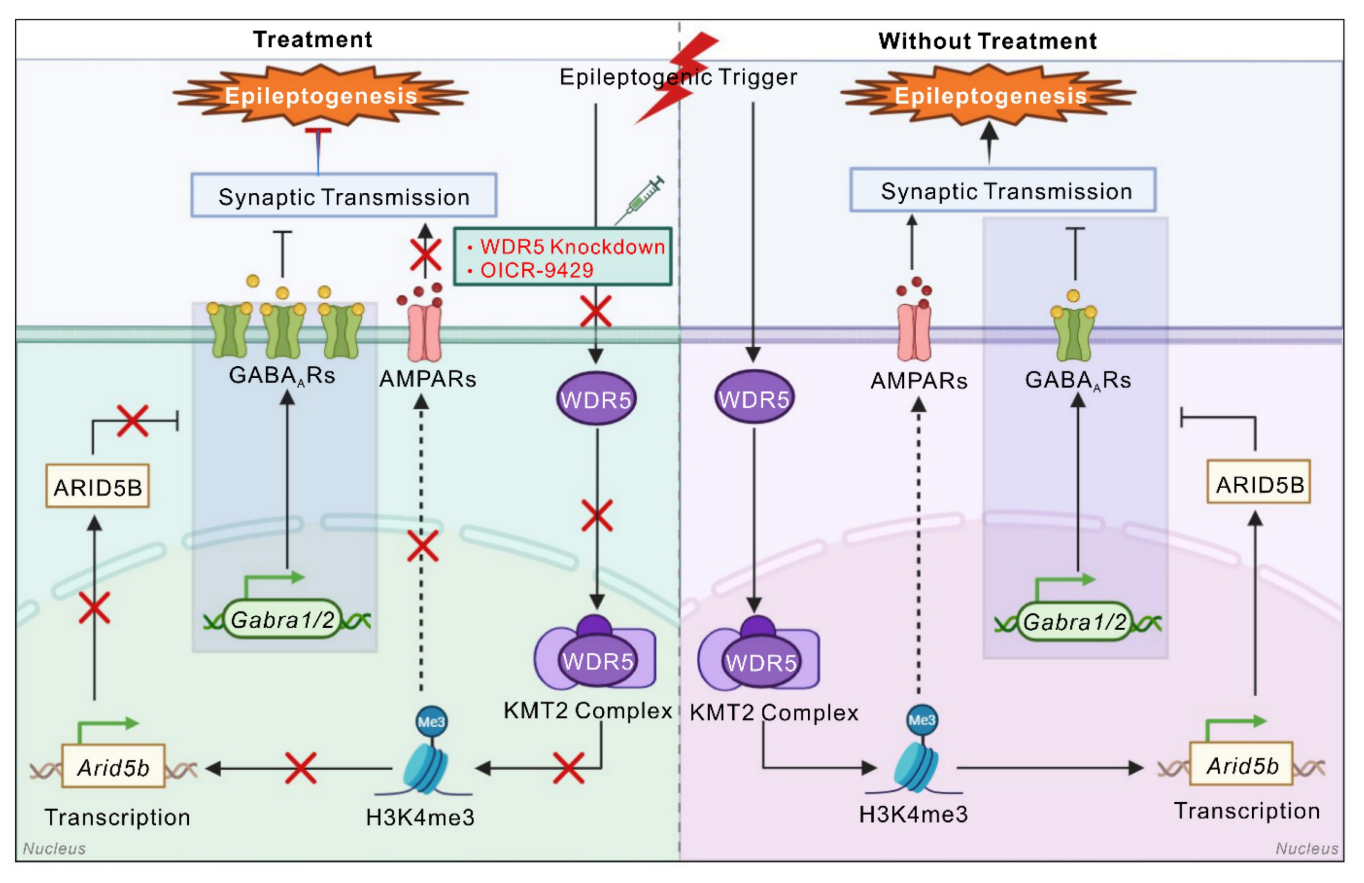
** A mechanistic model of the WDR5-H3K4me3-ARID5B axis in driving epileptogenesis.** During epileptogenesis, the neuronal WDR5-KMT2 complex promotes the transcription of the *Arid5b* gene by increasing H3K4me3 modification. The upregulated ARID5B protein subsequently suppresses the expression of GABAergic receptor subunits, impairing inhibitory synaptic transmission and ultimately promoting epileptogenesis. In addition, the WDR5-H3K4me3 axis may directly or indirectly regulate genes involved in glutamatergic synaptic transmission in an ARID5B-independent manner. Pharmacological inhibition of the WDR5-KMT2 complex or genetic knockdown of WDR5 exerts significant anti-epileptogenic effects. This figure was created with BioRender.

In summary, our study characterizes a novel hippocampal neuron-specific mechanism whereby the WDR5-ARID5B epigenetic axis disrupts the E/I balance through GABA_A_R downregulation to drive epileptogenesis (Figure 9). These findings advance our understanding of epigenetic control in epilepsy pathogenesis. Furthermore, these results offer a basis for developing multitarget therapeutic strategies against epileptogenesis by targeting this epigenetic pathway.

## Conclusions

In summary, our results demonstrate that upregulation of the WDR5-KMT2 complex during epileptogenesis elevates global H3K4me3 levels. The neuronal WDR5-H3K4me3 axis exacerbates epileptic phenotypes through dual mechanisms: it increases H3K4me3 occupancy at the* Arid5b* promoter to drive its transcriptional activation, and it may directly or indirectly regulate glutamatergic synaptic transmission in an ARID5B-independent manner. Elevated ARID5B protein subsequently decreases GABA_A_R subunit expression, impairing inhibitory synaptic transmission and promoting epileptogenesis (Figure 9). These findings elucidate the pivotal role of the neuron-specific WDR5-H3K4me3 axis in epileptogenesis and provide a novel multitarget therapeutic strategy for refractory epilepsy.

## Supplementary Material

Supplementary figures and tables.

## Abbreviations

AAVs: adeno-associated viruses
AMPAR: α-amino-3-hydroxy-5-methyl-4-isoxazolepropionic acid receptor
AP: action potential
ARID5B: AT-rich interactive domain-containing protein 5B
ASH2L: Set1/Ash2 histone methyltransferase complex subunit ASH2
ASMs: antiseizure medications
ChIP-seq: chromatin immunoprecipitation sequencing
Co-IP: coimmunoprecipitation
DEGs: differentially expressed genes
DIV: day *in vitro*
DPY30: protein dpy-30 homolog
eGFP: enhanced green fluorescent protein
E/I: excitation-inhibition
GABA_A_R: γ-aminobutyric acid type A receptor
GFAP: glial fibrillary acidic protein
GO: Gene Ontology
GSEA: gene set enrichment analysis
GTCS: generalized tonic‒clonic seizure
H3K4me3: histone H3 lysine 4 trimethylation
hSyn: human synapsin 1
Iba1: induction of brown adipocytes 1
IHKA-TLE: intrahippocampal kainic acid-induced TLE
*Kcnj10*: potassium inwardly-rectifying channel, subfamily J, member 10
KDM5A: lysine-specific demethylases 5A
KDM5B: lysine-specific demethylases 5B
KDM5C: lysine-specific demethylases 5C
KDM5D: lysine-specific demethylases 5D
KEGG: Kyoto Encyclopedia of Genes and Genomes
KMT2: lysine methyltransferase 2
*Lactb2*: lactamase, beta 2
mAHP: medium afterhyperpolarization
MAP2: microtubule-associated protein 2
mEPSC: miniature excitatory postsynaptic current
mIPSC: miniature inhibitory postsynaptic currents
NMDAR: N-methyl-D-aspartate receptor
PPR: paired-pulse ratio
qPCR: quantitative real-time PCR
RBBP5: retinoblastoma-binding protein 5
RMP: resting membrane potential
RNA-seq: RNA sequencing
SE: status epilepticus
SRSs: spontaneous recurrent seizures
*Slc23a1*: solute carrier family 23 (nucleobase transporters), member 1
TLE: temporal lobe epilepsy
WB: western blot
WDR5: WD repeat-containing protein 5
