# Supplementary material for: Neuron-specific WDR5 epigenetically upregulates ARID5B to impair GABAergic synaptic transmission and promotes epileptogenesis
Source: Theranostics. 2026 Jan 1;16(6):2721–47. doi: 10.7150/thno.122246 (PMC12775672; doi:10.7150/thno.122246)
Supplement: Supplementary file 1 — Supplementary figures and tables. [file thnov16p2721s1.pdf]

Supplementary Information

Neuron-specific WDR5 epigenetically upregulates ARID5B to impair GABAergic synaptic transmission and promotes epileptogenesis

Juan Gu[1,2,3], Ping-Yang Ke[4], Xin-Yu Zhang[1], Chao Liu[1], Yuan Yang[1], Ming-Lan Yu[1], Zhen-Zhong Xu[3], Chun-Xiang Zhang[1✉], Wei Dong[1✉]

1. Key Laboratory of Medical Electrophysiology of Ministry of Education and Medical, Electrophysiological Key Laboratory of Sichuan Province, Institute of Cardiovascular Research, Southwest Medical University, Luzhou 646000, China
2. Department of Neurology, The Affiliated Hospital of Southwest Medical University, Luzhou 646000, China
3. Department of Anesthesiology, First Affiliated Hospital and School of Brain Science and Brain Medicine, Zhejiang University School of Medicine, Hangzhou 310058, China
4. Department of Neurology, Chongqing Key Laboratory of Major Neurological and Mental Disorders, Neurology Key Laboratory of Chongqing Education Commission of China, Chongqing Key Laboratory of Neurology, The First Affiliated Hospital of Chongqing Medical University, Chongqing 400016, China

✉ Corresponding authors: Wei Dong (dongwei@swmu.edu.cn); Chun-Xiang Zhang (zhangchunxiangKZL@163.com)

Figure S1-8

Table S1-2

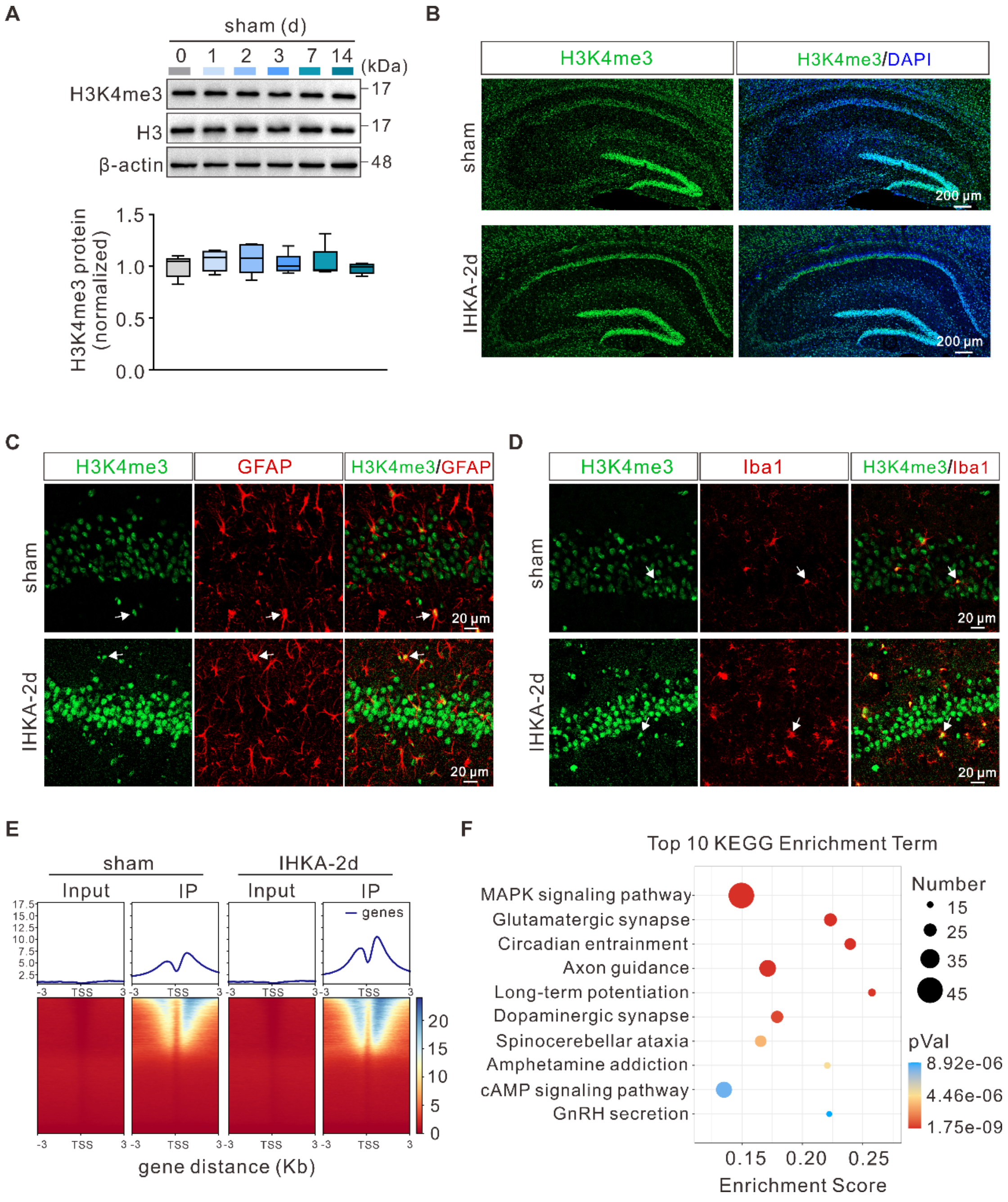

Figure S1. H3K4me3 protein expression and subcellular localization by immunoblot/immunofluorescence and genome-wide binding profiling by ChIP-seq. (A) Representative immunoblots (left) and quantitative analysis (right) of H3K4me3 in the ipsilateral hippocampus following sham surgery. β-actin was used as a loading control. n = 5 biologically independent mice per group. **(B)** Representative confocal images of the hippocampus immunostained for H3K4me3 (green) in Sham and IHKA-2d mice. Nuclei are counterstained with DAPI (blue). **(C)** Representative

confocal images of hippocampal CA1 from sham control and 2 d post-IHKA mice, immunostained for H3K4me3 (green) and glial fibrillary acidic protein (GFAP, red). **(D)** Representative confocal images of hippocampal CA1 from sham control and 2 d post-IHKA mice, immunostained for H3K4me3 (green) and ionized calcium-binding adapter molecule 1 (Iba1, red). **(E)** Heatmap visualization of H3K4me3 ChIP-seq signal density (±3 kb regions centered on TSS) in sham versus IHKA-2d mice. **(F)** KEGG pathway enrichment analysis of genes with differential H3K4me3 occupancy. Dot size reflects the gene count per pathway, and dot color indicates statistical significance (P values) calculated by the hypergeometric test. Statistical significance was determined by Kruskal-Wallis test with Dunn's post hoc test (Figure S1A).

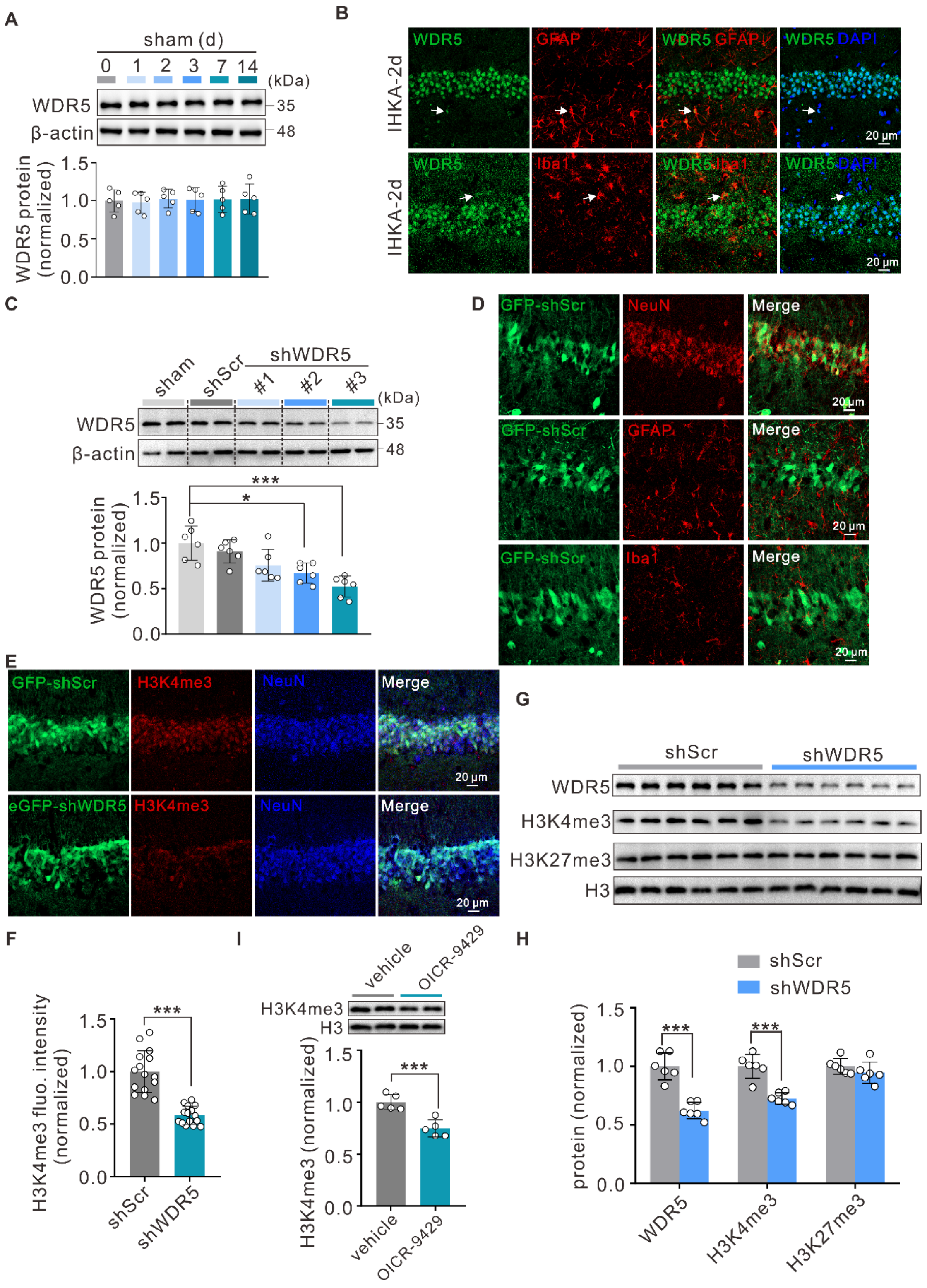

Figure S2. WDR5 expression patterns, subcellular localization, and regulatory effects on H3K4me3 and H3K27me3. (A) Representative immunoblots (top) and quantitative analysis (bottom) of WDR5 in the ipsilateral hippocampus following sham

surgery. n = 5 biologically independent mice per group. **(B)** Representative confocal images of the hippocampal CA1 from 2 d post-IHKA mice, stained for WDR5 (green), GFAP/Iba1 (red), and DAPI (blue). **(C)** Representative immunoblots (top) and quantitative analysis (bottom) of WDR5 protein levels in hippocampal lysates from mice transduced with indicated AAVs. n = 6 biologically independent mice per group. **(D)** Confocal microscopy validation of neuron-specific shScr transduction efficiency in hippocampal CA1. Sections from shScr transduced mice were co-stained with eGFP (green) and cell-type markers: NeuN (red), GFAP (red), or Iba1 (red). **(E)** Representative images of triple immunofluorescence staining. Coronal brain sections containing the hippocampal region from mice transfected with the indicated AAVs were co-stained with antibodies against eGFP (green), NeuN (blue), and H3K4me3 (red). **(F)** Quantitative analysis of H3K4me3 fluorescence intensity in hippocampal tissues transduced with neuron-specific AAVs for WDR5 knockdown or non-targeting control. n = 15 fields (shScr,3 mice), n = 15 fields (shWDR5, 3 mice). **(G-H)** Representative immunoblots (**G**) and quantitative analysis (**H**) of nuclear WDR5, H3K4me3, and H3K27me3 protein levels in hippocampal lysates from PTZ-kindled mice transduced with indicated AAVs. n = 6 biologically independent mice per group. $^{*}p < 0.05$, $^{***}p < 0.001$. **(I)** Representative immunoblots (top) and quantitative analysis (bottom) of H3K4me3 levels in nuclear fractions isolated from hippocampal tissues of normal mice receiving the indicated treatments. n = 5 biologically independent mice per group. Statistical analyses were performed as follows: one-way ANOVA with Dunnett's post hoc test (Figure S2A, C); two-tailed unpaired Student’s t-tests (Figure S2F, I); two-tailed unpaired Student’s t-tests with Holm-Šidák correction for multiple comparisons (Figure S2H).

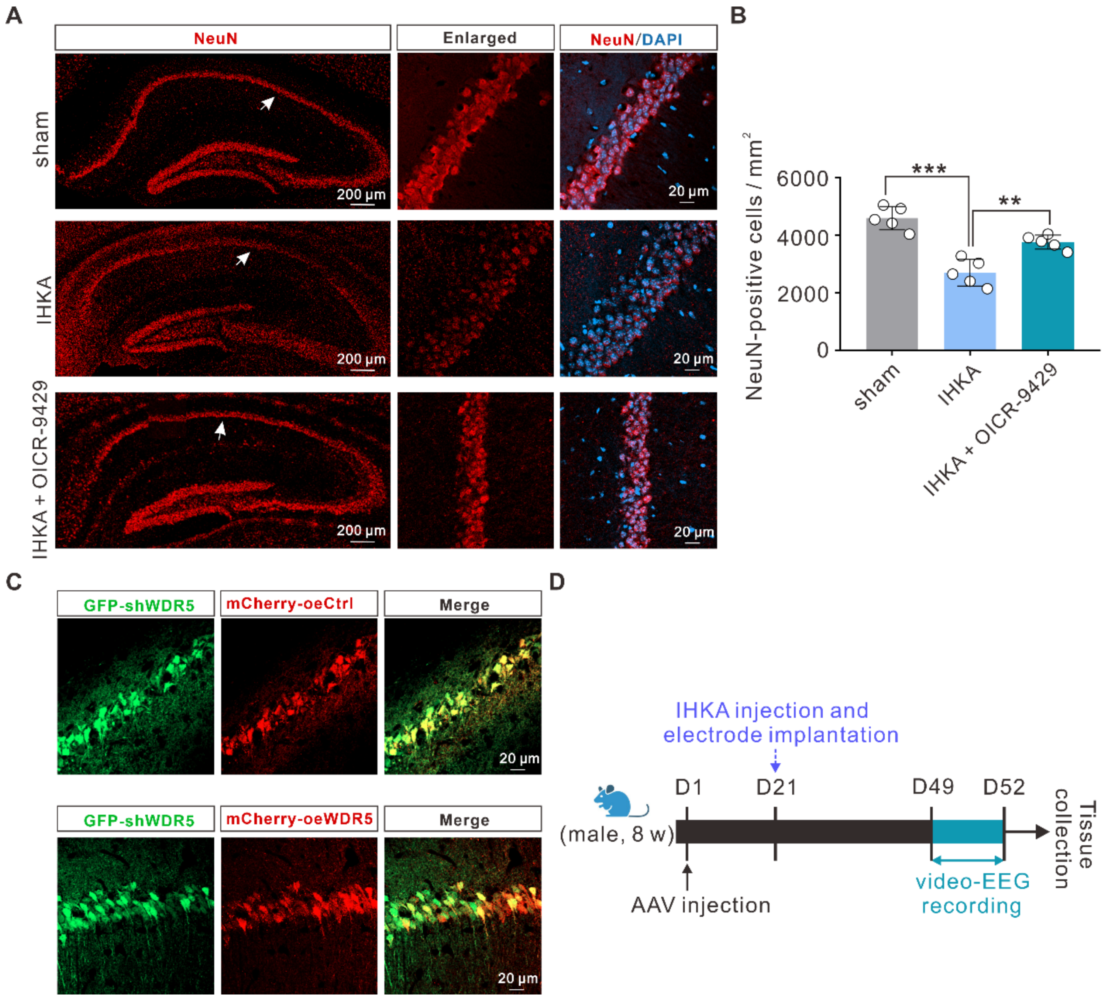

Figure S3. Assessment of OICR-9429 neuroprotection, AAV transduction efficiency, and behavioral study workflow. (A) Representative NeuN immunofluorescence staining images of the hippocampal CA1 region from mice across different treatment groups, collected at 30 days post-IHKA injection. **(B)** Quantitative statistical analysis of the number of NeuN-positive cells in the CA1 region under conditions shown in (A). n = 5 biologically independent mice per group. **(C)** Validation of neuron-specific AAVs transduction in hippocampal CA1. Confocal images show shWDR5-eGFP (green) and oeWDR5-mCherry/oeCtrl-mCherry (red) co-expression. **(D)** Schematic diagram of experimental design and timeline. Statistical significance was determined by one-way ANOVA with Tukey's post hoc test (Figure S3B).

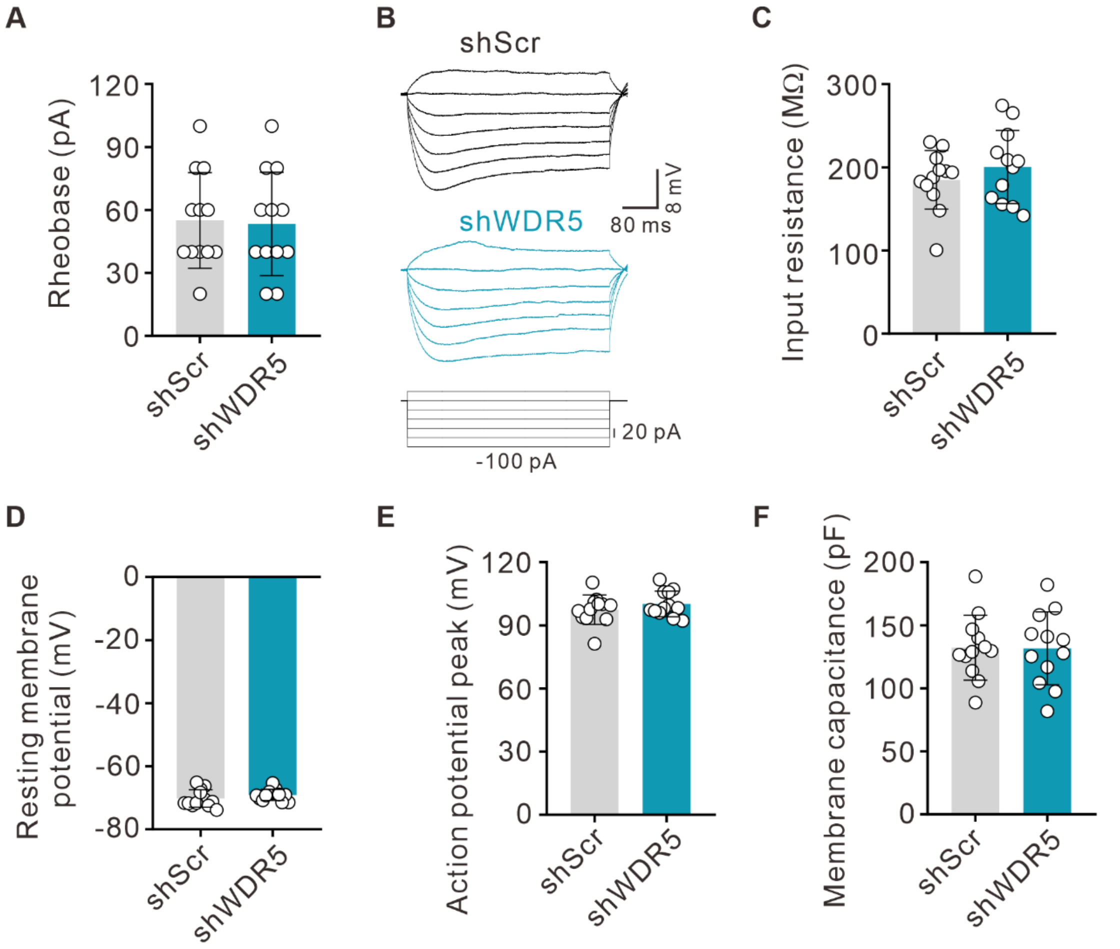

Figure S4. WDR5 knockdown does not affect intrinsic properties of hippocampal CA1 pyramidal neurons in IHKA-2d mice. (A) Rheobase quantification in CA1 pyramidal neurons. Neurons were recorded from IHKA-2d mice transduced with shScr (n = 12 cells/4 mice) or shWDR5 (n = 12 cells/4 mice). Rheobase was defined as the minimal current injection (500-ms duration) required to elicit at least one action potential. **(B)** Representative traces of voltage responses to a depolarizing from −100 to 20 ρA current step in CA1 pyramidal neurons from IHKA-2d mice transduced with shScr or shWDR5. **(C-F)** Input resistance (**C**), resting membrane potential (**D**), AP peak amplitude (**E**), and membrane capacitance (**F**) quantification in CA1 pyramidal neurons from IHKA-2d mice transduced with shScr **(**n = 12 cells/4 mice) or shWDR5 **(**n = 12 cells/4 mice). Statistical significance was determined by two-tailed unpaired Student’s t-tests (Figure S4A, C-F).

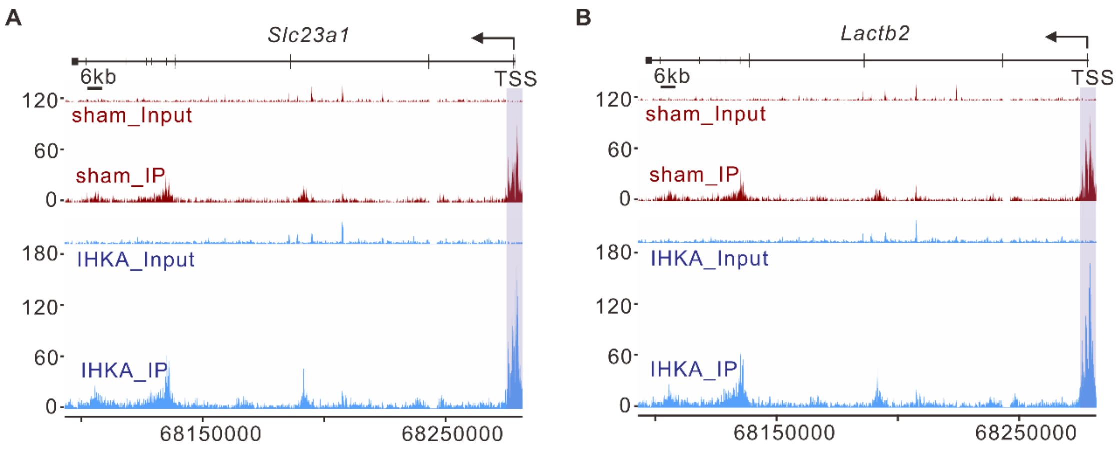

Figure S5. Genome browser snapshots of H3K4me3 ChIP-seq signals at the *Slc23a1* and *Lactb2* loci. (A) Genome browser snapshots of H3K4me3 ChIP-seq signals at the *Slc23a1* locus in sham control versus IHKA-2d groups. Purple shading highlights H3K4me3 peak enrichment at the *Slc23a1* promoter region. **(B)** Genome browser snapshots of H3K4me3 ChIP-seq signals at the *Lactb2* locus in sham control versus IHKA-2d groups. Purple shading highlights H3K4me3 peak enrichment at the *Lactb2* promoter region.

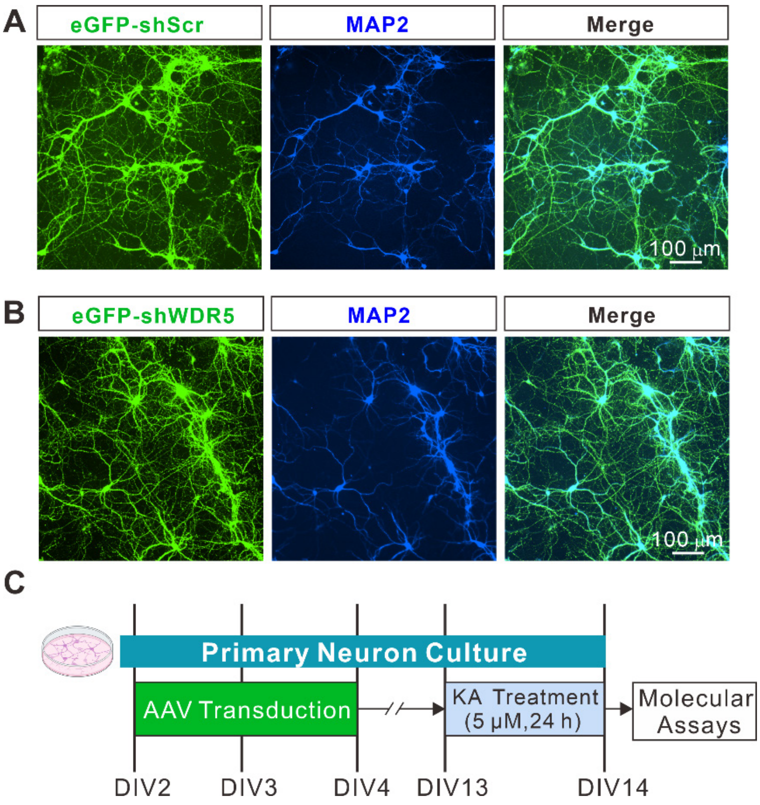

Figure S6. AAV-mediated eGFP expression in primary hippocampal neurons. (A-B) Confocal images of AAV-transduced primary hippocampal neurons at DIV14, showing efficient eGFP reporter expression (green) in MAP2-immunopositive neurons (blue) in the shScr (**A**) and shWDR5 (**B**) groups. **(C)** Schematic timeline of the in vitro KA model establishment and AAV transduction in primary hippocampal neurons. Primary neurons were cultured and transduced with AAVs at 2-3 days in vitro (DIV). To establish the KA model, neurons were treated with 5 µM KA for 24 hours, starting at DIV13. All molecular biological assays were performed at DIV14.

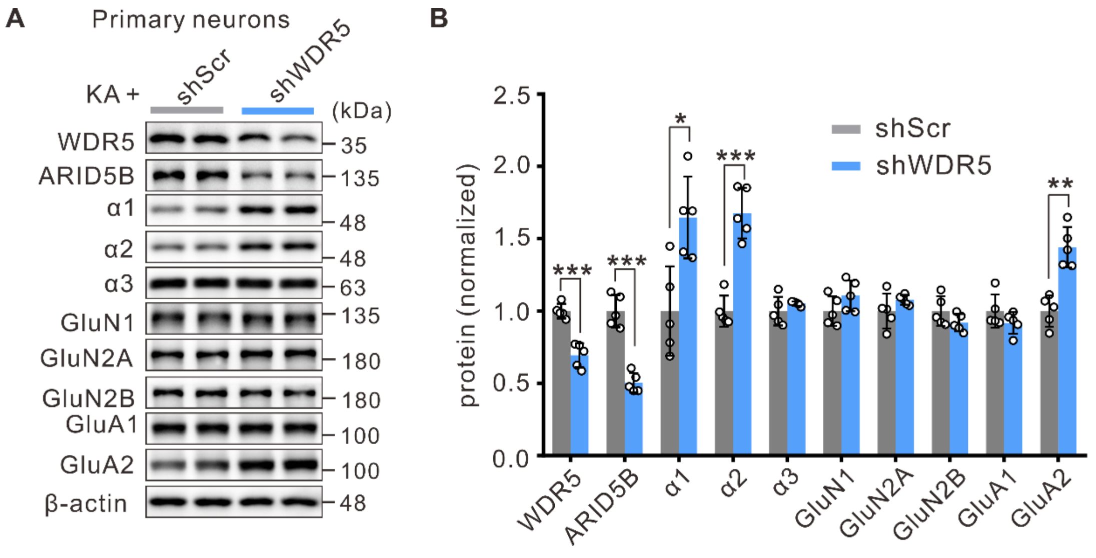

Figure S7. WDR5 knockdown modifies GABA/glutamate receptor subunit expression in cultured hippocampal neurons. (A-B) Representative immunoblots (**A**) and quantitative analysis (**B**) of WDR5, ARID5B and other synaptic proteins in cell lysates prepared from KA-treated primary cultured hippocampus neurons transfected with shScr or shWDR5. n = 5 biologically independent cultures per group. *p < 0.05, **p < 0.01, ***p < 0.001. Statistical comparisons were performed using two-tailed unpaired Student's t-tests with Holm-Šidák correction for multiple comparisons.

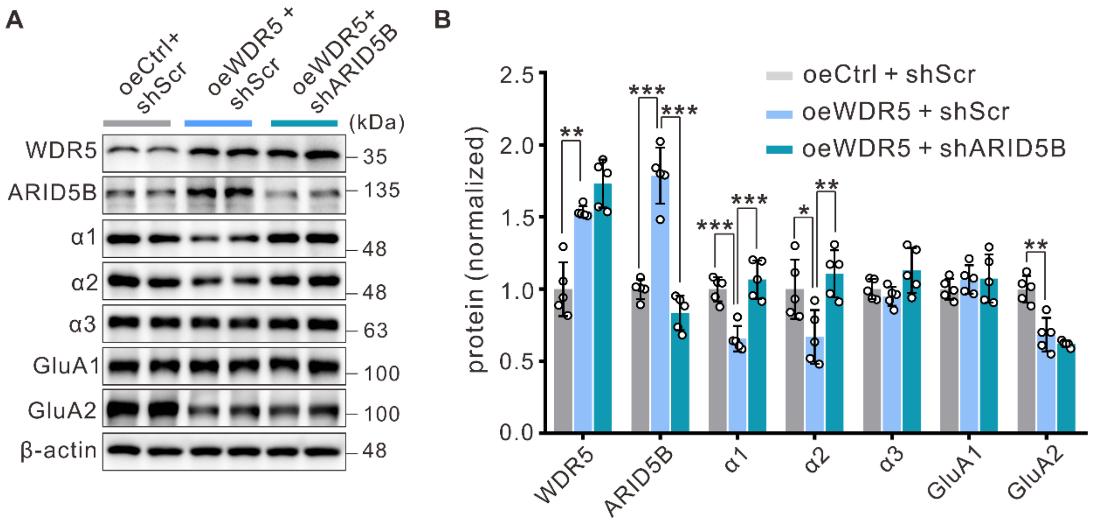

Figure S8. WDR5 overexpression-induced reduction in GABA$_A$R α1 and α2 subunits is rescued by ARID5B knockdown. (A-B) Representative immunoblots (**A**) and quantitative analysis (**B**) of WDR5, ARID5B and other synaptic proteins in hippocampal lysates from IHKA-TLE mice transduced with indicated AAVs. n = 5 biologically independent mice per group. *$p < 0.05$, **$p < 0.01$, ***$p < 0.001$ (Welch's ANOVA with Games-Howell post hoc tests for WDR5 and GluA2; one-way ANOVA with Tukey's post hoc test for other proteins).

Table S1. Details about antibodies.

Antibody target	Species	Supplier, catalog number	Working concentration (application)
ARID5B	rabbit	Novus, NBP3-13006/NBP1-83622	1:1000 (WB), 1:200 (IF)
Beta Actin	rabbit	Proteintech, 20536-1-AP	1:5000 (WB)
GABA-A receptor α1	rabbit	Proteintech, 12410-1-AP	1:2000 (WB)
GABA-A receptor α1 extracellular	rabbit	SYSY, 224203	1:400 (IF)
GABA-A receptor α2	rabbit	Abcam, ab307359	1:1000 (WB)
GABA-A receptor α2 extracellular	rabbit	SYSY, 224103	1:400 (IF)
GABA-A receptor α3	rabbit	Abcam, ab316969	1:1000 (WB)
GFAP	mouse	Cell Signaling Technology, 3670	1:400 (IF)
GFP tag	rabbit	Proteintech, 50430-2-AP	1:200 (IF)
GluA1	mouse	Proteintech, 67642-1-Ig	1:20000 (WB)
GluA2	rabbit	Proteintech, 11994-1-AP	1:1000 (WB)
GluN1	rabbit	Proteintech, 27676-1-AP	1:1000 (WB)
GluN2A	rabbit	Proteintech, 28525-1-AP	1:1000 (WB)
GluN2B	rabbit	Proteintech, 21920-1-AP	1:1000 (WB)
Histone-H3	rabbit	Proteintech, 17168-1-AP	1:5000 (WB)
Iba1	mouse	Abcam, ab283319	1:100 (IF)
MAP2	Guinea pig	SYSY, 188004	1:400 (IF)
Mono-Methyl-Histone H3 (Lys4)	rabbit	Cell Signaling Technology, 5326	1:1000 (WB)
NeuN	mouse	Proteintech, 66836-1-Ig	1:200 (IF)
Normal Rabbit IgG	rabbit	Cell Signaling Technology, 2729	1:100 (IP)

Tri-Methyl-Histone H3 (Lys4)	rabbit	Cell Signaling Technology,	1:1000 (WB), 2.5μg (ChIP), 1:200 (IF)
Tri-Methyl-Histone H3 (Lys9)	rabbit	Cell Signaling Technology, 13969	1:1000 (WB)
Tri-Methyl-Histone H3 (Lys27)	rabbit	Cell Signaling Technology, 9733	1:1000 (WB)
KMT2A (MLL1)	rabbit	Cell Signaling Technology, 14197	1:1000 (WB)
SETD1A	rabbit	Cell Signaling Technology, 50805	1:1000 (WB)
SETD1B	rabbit	Abcam, ab300479	1:1000 (WB)
Sodium Potassium ATPase	rabbit	Abcam, ab76020	1:20000 (WB)
WDR5	rabbit	Abcam, ab307664	1:1000 (WB), 1:30 (IP), IF (1:200)
WDR5	rabbit	Invitrogen, MA5-32760	1:1000 (WB), 3 μg (ChIP)
HRP-conjugated Affinipure Goat Anti-Mouse IgG(H+L)	mouse	Proteintech, SA00001-1	1:5000 (WB)
HRP-conjugated Affinipure Goat Anti-Rabbit IgG(H+L)	rabbit	Proteintech, SA00001-2	1:5000 (WB)
DyLight 405-conjugated AffiniPure Goat Anti-Guinea Pig IgG (H+L)	guinea pig	Jackson ImmunoResearch, 147530	IF (1:400)
Dylight 488, Goat Anti-Rabbit IgG	rabbit	Abbkine, A23220	IF (1:200)

Dylight 594, Goat Anti-Mouse IgG	mouse	Abbkine, A23410	IF (1:200)
Dylight 594, Goat Anti-Rabbit IgG	rabbit	Abbkine, A23420	IF (1:200)

Abbreviations: ChIP, Chromatin immunoprecipitation; IF, immunofluorescence; IP, immunoprecipitation; WB, western blot.

Table S2. Primers used for qPCR and ChIP-qPCR.

Gene		Sequences (5'- -3')	Uses
Actb	Forward	GGCTGTATTCCCCTCCATCG	qPCR
	Reverse	CCAGTTGGTAACAATGCCATGT	
Arid5b	Forward	TCGCAGACACTACGAAAGGTT	
	Reverse	ATTGGCGGCAGAGGCTTATC	
Kdm5a	Forward	CAGCCACCAGTGTCTCAACG	
	Reverse	AGTCTTCCGTGTGTCATCAGC	
Kdm5b	Forward	TGGAGCTCTTGCTTTAGTCGG	
	Reverse	GTCTGCACGGGTTTCGGAC	
Kdm5c	Forward	GGCTCGAAGACCGGACAC	
	Reverse	GTCTGGAGCCAACCTTGTGT	
Kdm5d	Forward	CTCTTTGCCCGTCGTCCATA	
	Reverse	TCGCTAGCAGCAGAACACTT	
Kmt2a	Forward	TCCAACAGGTAGCCAATCG	
	Reverse	GCTGACCACAAAGCACAGTTCAC	
Kmt2b	Forward	GGAAGCCAGATGAAAGGACTCC	
	Reverse	TGGTCCAAGGATGGAGGCAACA	
Kmt2c	Forward	ACTGTGAGCTGTGGGTACAT	
	Reverse	CCAAACTCGGCAGGACAAAT	
Kmt2d	Forward	TCCCGAATCAAACAGGGTCG	
	Reverse	CAGCCACCAGTGTCTCAACG	
Lactb2	Forward	TCCTAGGAGAAGGGACGACA	
	Reverse	CAGGAGTTGTTGTGTAGACTTGT	
Setd1a	Forward	AAGGGATTGACTGCAGCCTC	
	Reverse	GAGTACGACCCACGACCTTC	
Setd1b	Forward	CATGGACACCCAGGGTATGAG	
	Reverse	GTTGAACTTGAGCAGGTCGC	
Slc23a1	Forward	GGTGCTATCAACACAGGCAT	

	Reverse	TGGCTGACGTCTCACTGTTG	
Arid5b	Forward	CTGAATTCTGAGGGCCCAA	ChIP-qPCR
	Reverse	GGCTACTTTCACTTGTACACATAAC	
